# Synthesis of Melatonin Derivatives and the Neuroprotective Effects on Parkinson’s Disease Models of *Caenorhabditis elegans*

**DOI:** 10.3389/fchem.2022.918116

**Published:** 2022-06-08

**Authors:** Li He, Jing-Jing Du, Jun-Jie Zhou, Meng-Ting Chen, Lu Luo, Bao-Qiong Li, Xiang-Zhi Zhang, Wen-Zhe Ma, Ai-Jun Ma, Na Feng

**Affiliations:** ^1^ School of Biotechnology and Health Sciences, Wuyi University, Jiangmen, China; ^2^ State Key Laboratory of Quality Research in Chinese Medicine, Macau University of Science and Technology, Macau, China

**Keywords:** derivative of melatonin, synthesis, antioxidant activity, *C. elegans*, Parkinson’s disease

## Abstract

Melatonin (MT) is a hormone with antioxidant activity secreted by the pineal gland in the human brain, which is highly efficient in scavenging free radicals and plays an important role in the neuro-immuno-endocrine system. Emerging evidence showed that MT supplementation was a potential therapeutic strategy for Parkinson’s disease (PD), which inhibits pathways associated with oxidative stress in PD. In this study, we reported a C7-selective olefination of melatonin under rhodium catalysis with the aid of P^III^-directing groups and synthesized 10 new melatonin-C7-cinnamic acid derivatives (6a–6j). The antioxidant potential of the compounds was evaluated both by ABTS and ORAC methods. Among these newly synthesized melatonin derivatives, 6a showed significantly higher activity than MT at 10^−5^ M. In the transgenic *Caenorhabditis elegans* model of PD, 6a significantly reduces alpha-synuclein aggregation and dopaminergic neuronal damage in nematodes while reducing intracellular ROS levels and recovers behavioral dysfunction induced by dopaminergic neurodegeneration. Further study of the mechanism of action of this compound can provide new therapeutic ideas and treatment strategies for PD.

## Introduction

Melatonin (MT), secreted by the midbrain pineal gland and some peripheral tissues, is a tryptophan metabolite that activates multiple intracellular signaling pathways (Pohanka et al., 2011) and has a variety of physiological effects, such as regulating circadian rhythms, scavenging free radicals, enhancing immunity, and inhibiting oxidation of biomolecules (Yu et al., 2000; Skene and Swaab., 2003; Wu et al., 2004). In addition, melatonin has protective effects against neurodegenerative diseases (Rudnitskaya et al., 2015).

Parkinson’s disease (PD) is a neurodegenerative disease with motor and non-motor symptoms characterized by the loss of dopaminergic neurons in the nigrostriatal and the formation of Lewy body proteins (Calabrese et al., 2018; Bouca-Machado et al., 2019). The main features of PD are resting tremor, rigidity, and motor retardation (Mann and Yates, 1982; Smeyne and Jackson-Lewis, 2005; Jankovic and Stacy., 2007; Hirsch et al., 2012). This may be due to the selective loss of dopaminergic (DA) neurons in the substantia nigra densa (SN), resulting in neurological dysfunction (Delenclos et al., 2016). Pathologically, PD is also characterized by the formation of alpha-synuclein (α-syn) aggregates. The pathological process of PD involves multiple pathways, including apoptosis, autophagy, oxidative stress, *α*-syn aggregation, and alterations in neurotransmitters (Olzmann et al., 2010; Martinez et al., 2017; Aditi and Verma, 2019; Brunetti et al., 2020).

There is emerging evidence supporting the impact of oxidative stress on PD, and therefore, drugs with antioxidant activity are expected to be a potential treatment for PD (Moosmann and Behl, 2002; Abou-Sleiman et al., 2006; Weber and Ernst, 2006; Sayre et al., 2008). Studies suggest that MT supplementation is a therapeutic approach for PD disorder. The use of MT inhibits a number of pathways associated with oxidative stress response, *α*-syn aggregation, and dopamine loss in PD (Chen et al., 2002; Zarranz et al., 2004; Berendse et al., 2010). MT may also improve some non-motor symptoms in PD patients.

The nematode *Caenorhabditis elegans* (*C. elegans*) is a powerful genetic model system for exploring PD and related molecular mechanisms (Nass and Chen, 2008; Anand et al., 2020). In this study, we used two well-established *C. elegans* models to assess the anti-Parkinsonian effects of melatonin derivatives and to explore their associated potential neuroprotective mechanisms.

## Materials and Methods

### Materials and Strains

^1^H NMR and ^13^C NMR spectra were both performed on a 500 MHz Bruker NMR spectrometer using TMS as an internal standard (chloroform-*d* as the solvent). Mass spectra were carried out using a Thermo Fisher LCQ Fleet LC-MS mass spectrometer. All reagents used were of analytical grade.

Strains: Bristol N2: wild-type N2 nematode; NL5901: pkIs2386 [unc-54p:α-synuclein:YFP + unc-119 (+)], a transgenic nematode model of PD disorder expressing yellow fluorescent protein-tagged human *α*-synuclein in muscle; BZ555: egIs1 [dat-1p:GFP], pharmacological model of PD disorder nematode, dat-1 encodes a plasma membrane dopamine transporter protein labelled with a green fluorescent mono-white that can be observed as bright green fluorescence at the junctions between dopamine neuronal soma cells, purchased from the CGC (*Caenorhabditis* Genetics Center); *E. coli* OP50: *Escherichia coli* OP50, uracil leakage mutant strain, used as normal food for feeding *C. elegans*.

### Synthesis of 6a–6j

Our group designed a P^III^-directing group (*N*-P*t*Bu_2_), which directed C7-H functionalization of melatonin with olefins through rhodium catalysis. (Borah and Shi, 2018). We describe the development of the decarbonylative cross-couplings (Zhao and Yu, 2008; Lei et al., 2015) of carboxylic acids with melatonin at the C7 position by P^III^-chelation-assisted Rh^I^-catalyzed C-C bond activation (Qiu et al., 2019).

The decarbonylative reaction was further optimized with substituted cinnamic acid 4 as a partner to produce the melatonin C7-olefination products. We found that the reaction achieved optimal efficiency and selectivity with 2.5 equiv. of Boc_2_O in the presence of 5.0 mol% Rh(cod)_2_OTf as a catalyst. Product 5 was obtained after reaction at 120°C for 18 h under a nitrogen atmosphere. Furthermore, the directing group (P*t*Bu_2_) could be easily removed by TBAF in THF, and N-free melatonin derivatives 6 were isolated at the yields of 47–67% (Han et al., 2019; Qiu et al., 2019) Scheme 1.

**SCHEME 1:**
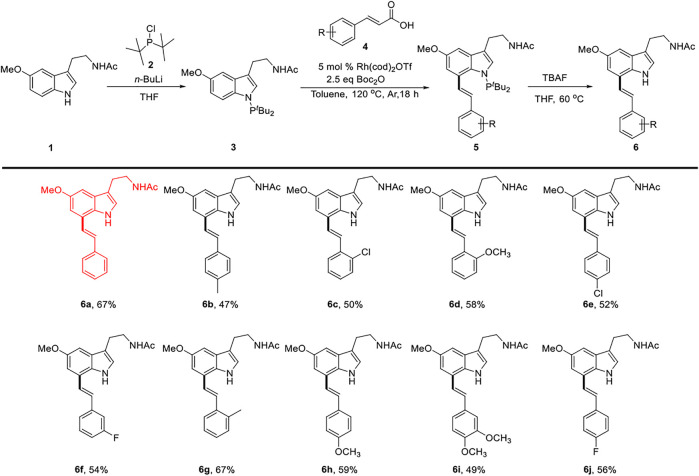
Synthesis route for compounds 6a∼ 6j.

### N-(2-(5-Methoxy-7-Styryl-1h-Indol-3-yl)ethyl)acetamide (6a)

Light yellow oil. 67% yield. ^1^H NMR (500 MHz, Chloroform-*d*) *δ*: 8.47 (s, 1H), 7.60∼7.49 (m, 2H), 7.44∼7.35 (m, 2H), 7.35∼7.27 (m, 2H), 7.18 (s, 1H), 7.09 (t, *J* = 2.0 Hz, 2H), 7.03 (d, *J* = 2.3 Hz, 1H), 5.62 (s, 1H), 3.92 (s, 3H), 3.62 (q, *J* = 6.5 Hz, 2H), 2.97 (t, *J* = 6.8 Hz, 2H), and 1.94 (s, 3H); ^13^C NMR (125 MHz, chloroform-*d*) *δ*: 170.2, 154.4, 137.2, 130.5, 129.8, 128.8, 128.5, 127.9, 126.5, 124.3, 122.9, 122.3, 113.2, 109.9, 100.7, 56.1, 39.8, 25.3, and 23.4; LC-MS (ESI) *m/z*: calcd. for C_21_H_22_N_2_O_2_ {[M + Na]^+^} 357.1573, found 357.1572.

The details of compounds 6b–6j can be found in the Supplementary Material S1.

### ABTS Method

The ABTS assay was performed in accordance with a previously reported procedure (Schaich et al., 2015; Sommer et al., 2022). A volume of 5 ml of 7 mmol/L ABTS and 88 μL of 140 mmol/L potassium persulfate were mixed and left for 12 h at room temperature in the dark to form an ABTS^+^ free radical reserve solution. The reserve liquid was relatively stable at room temperature away from the light. Before use, it was diluted into the working liquid with ultra-pure water, and its absorbance was required to be 0.7 ± 0.02 at 30°C and a wavelength of 734 nm. The samples were prepared with DMSO into 5, 10, 20, and 40 μg/ml sample solutions for later use. During the determination, 100 μL of ABTS working solution was added to each well of the 96-well microtitration plate, and then 100 μL of sample solution at different concentrations was added to shake and mix. After 10 min, the absorbance at 734 nm was determined, and 100 μL of ABTS working solution mixed with 100 μL of DMSO was used to determine the blank absorbance A_0_. The absorbance of a 100 μL sample mixed with 100 μL of DMSO was defined as Ar. For each determination, experiments were performed in triplicate.

The radical scavenging rate of ABTS is calculated using the following formula: ABTS free radical scavenging rate (%) = [1 − (At − Ar)/A_0_] × 100%.

### ORAC Method

The ORAC method was performed as described previously (Huang et al., 2002; Ou et al., 2002) with slight modification. The reaction was carried out in 75 mM phosphate buffer (pH 7.4), while the addition of antioxidant substances produced more stable fluorescent signal that could reflect the antioxidant capacity.

A volume of 50 µL of samples to be tested or different concentrations of Trolox working solution (0.10, 0.08, 0.06, 0.04, and 0.02 mmol/L) were added to a 96-well plate, followed by the addition of 100 µL of FL working solution (8.4 × 10^–8^ mol/L) and shaking for 30 s. The fluorescence value F0 was recorded immediately, and then the reaction was shaken for 3 min and incubated at 37 °C for 10 min. A volume of 50 µL of AAPH working fluid was added to induce the reaction. The excitation wavelength and emission wavelength were 485 and 535 nm, respectively, and the fluorescence value Fn was recorded every 2.5 min. The reaction was considered to be over when the fluorescence decrease slowed. The initial fluorescence value (F0) is the pore fluorescence intensity reading without AAPH. fn = fluorescence value (Fn)/initial fluorescence value (F0); AUC = 2 × (f0 + f1 +...+ fn) − f0 − fn; NetAUC (ORAC value) = AUC_sample_ − AUC_AAPH_^+^. NetAUC was used to determine the capacity for absorbing free radicals. Here, AUC is the area under the fluorescence decay curve, and NetAUC is the protected area. In addition to the tested sample group, a blank group and an AAPH + group (for which 50 µL of 75 mM phosphate-buffered saline (PBS) solution was used as the sample substitution) were also tested. To create a standard curve, different concentrations of Trolox solution were used for the abscissa and NetAUC was used for the ordinate, and the absorption capacity of the sample was expressed as μmol TE/g DW. For each determination, experiments were performed in triplicate.

### Measurement of Reactive Oxygen Species (ROS)

2′,7′-Dichlorofluorescein diacetate (H_2_DCFDA), a universal oxidative stress indicator, was used as a probe for the detection of intracellular ROS. H_2_DCFDA was oxidized by ROS to form the fluorescent dye dichlorofluorescein (DCF) (Jia et al., 2021). The fluorescence intensity was proportional to the ROS level in *C. elegans*.

Briefly, age-matched L3 larvae were treated with 10 μM MT or **6a** for 24 h. Subsequently, 2000 nematodes per group were collected and washed three times with M9 buffer. The nematodes were suspended in 400 µL of PBS with 1% Tween-20 and homogenized to obtain worm lysate. The protein concentration of the lysate was determined using the BCA protein assay kit. A volume of 50 μL of nematode lysate was transferred into a 96-well black microplate and incubated with 50 μL of 50 μM H_2_DCFDA. Fluorescence intensity (485 nm excitation and 590 nm emission) was monitored using an enzyme plate reader (Wu et at., 2006; Liu et al., 2015; Zhao et al., 2018).

### α-Synuclein Accumulation Assay

PD pathology usually showed an accumulation of the *α*-syn. We used strain NL5901 to test the effect of MT and 6a on *α*-syn aggregation. The synchronized nematodes were immersed in M9 buffer containing 10 µM melatonin and 6a for 48 h at 20°C. L1 worms were transferred to OP50/NGM plates at 20°C for 65 h (L3 stage), and then the nematodes were transferred to plates containing OP50/NGM/5-fluorodeoxyuridine (FUDR, 0.04 mg/ml) and incubated at 20°C for 3 days. The young adults were washed three times with M9 buffer, then anesthetized with 10% sodium azide, and fixed on slides. The worms were observed using an Olympus BX63 fluorescent microscope to monitor the YFP expression (α-syn aggregation). The fluorescence intensity of each nematode was quantified using ImageJ software (Jadiya et al., 2011; Govindan et al., 2018; Anjaneyulu et al., 2020).

### 6-OHDA-Induced Damage to Dopaminergic Neurons

Dopaminergic neuron degeneration was induced by 50 mM 6-OHDA in *C. elegans* as described previously (Tucci et al., 2011). Briefly, the synchronized nematodes were immersed in M9 buffer containing MT or 6a for 48 h at 20°C, and then L1 worms were transferred to OP50/NGM plates at 20°C for 65 h (L3 stage), after which they were exposed to 50 mM 6-OHDA for 1 h. After exposure, the worms were washed with M9 buffer and transferred to OP50/NGM plates containing 0.04 mg/ml FUDR for 3 days at 20 °C for various assays (Chalorak et al., 2021; Ma et al., 2021).

### Behavior Assay

In general, N_2_ nematodes were treated with 50 mM 6-OHDA to induce degenerative lesions in dopamine neurons.

For thrashing assay, synchronized L4 stage nematodes were transferred to a 3-cm diameter Petri dish, 1 ml of M9 buffer was added, and after the nematodes were stabilized for 30 s, a Leica M205 FA microscope was used to continuously capture the worms for 10 s. The number of thrashing of the nematodes was counted (Lee et al., 2021).

For travel distance assay, nematodes of the synchronized L4 stage were transferred from food-containing dishes to non-food dishes and washed three times repeatedly in M9 buffer to remove the remaining food. The distance (mm) and speed (µm/sec) of the nematode’s movement in 20 s were calculated using the Leica M205 FA microscope and finally analyzed using GraphPad Prism software (Sawin et al., 2000).

### Statistical Analysis

All the experiments were performed in triplicate. The significance of differences between control and treated groups was analyzed by one-way analysis of variance (ANOVA), followed by Bonferroni’s method. *p*-values < 0.05 were accepted as statistically significant. Graphs were constructed using GraphPad Prism version 8.00.

## Results and Discussion

### Antioxidant Activity of Melatonin Derivatives *In Vitro*

**FIGURE 1:**
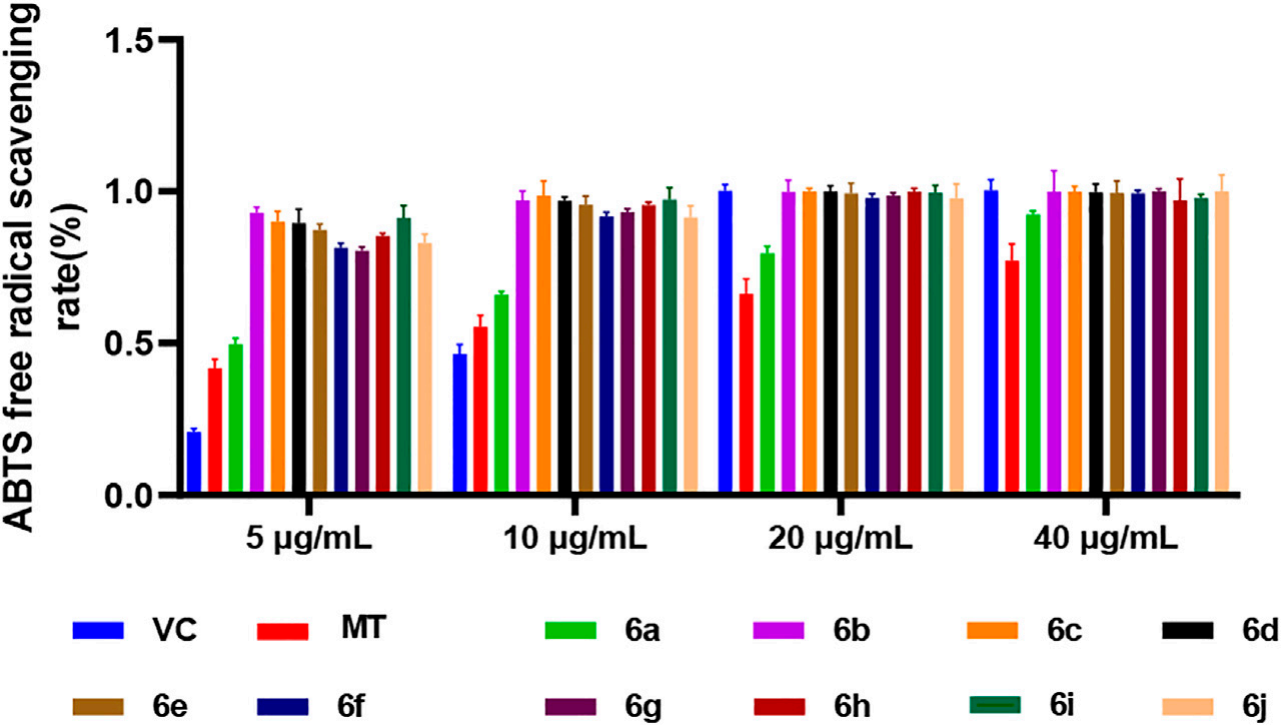
ABTS method for measuring the antioxidant activity of 6a∼ 6j.

In the ABTS method, the antioxidant activity of the 10 derivatives of melatonin at the C7 position, 6a–6j, increased in a concentration-dependent manner, and the antioxidant activity of 6a–6j was higher than that of melatonin at the same concentration, while the ABTS radical scavenging rate of the 10 derivatives, 6a–6j, was higher than that of vitamin C (VC) and MT at low concentrations of 5 and 10 μg/ml (Figure 1). These results indicated that the derivatization of melatonin at the C7 position could improve its antioxidant activity.

**FIGURE 2:**
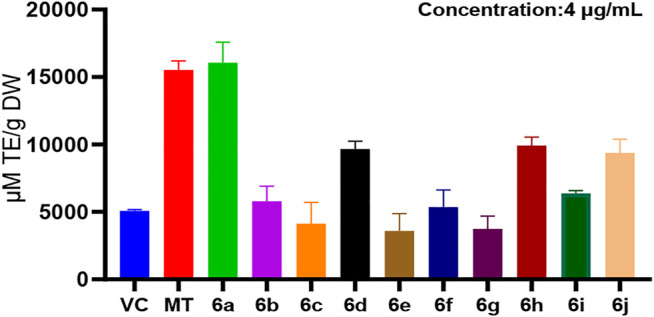
ORAC method for measuring the antioxidant activity of 6a∼ 6j.

In the ORAC method, at the same concentration (4 μg/ml), the antioxidant activity of derivatives 6b–6j at the C7 position of melatonin was lower than that of melatonin, whereas the antioxidant activity of 6a was higher than that of MT (Figure 2). This suggested that the derivatization of melatonin at the C7 position may increase its antioxidant activity.

In this study, the antioxidant activity of melatonin derivatives was assessed by two *in vitro* assays, the ABTS method and the ORAC method. Comparing the results of these two methods, we found that the antioxidant activity of 6a, a derivative of melatonin at position C7, was higher than that of MT and the positive control VC as well.

### 6a Decreased the ROS Level in *C. elegans*

**FIGURE 3:**
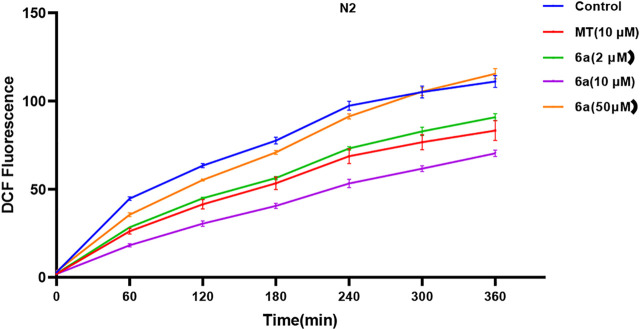
Effect of 6a on the ROS level in wild-type.

To further confirm the antioxidant effect of 6a, we tested the intracellular ROS level in *C. elegans*. We first examined the effect of 6a on the ROS levels in wild-type N2 nematodes. As shown in Figure 3, compared with the untreated group, after treatment with 6a at different concentrations (2, 10, and 50 µM), the levels of ROS in the nematodes decreased, and 10 µM of 6a achieved a significant decrease compared with the level in the untreated group (*p* < 0.001). Additionally, the ROS levels clearly declined in the 6a group compared with those in the MT group (*p* < 0.05). These results are consistent with the *in vitro* antioxidant assay, further demonstrating the antioxidant capacity of 6a. The excellent antioxidant activity of 6a suggests its potential as a therapeutic agent for PD disorders.

**FIGURE 4:**
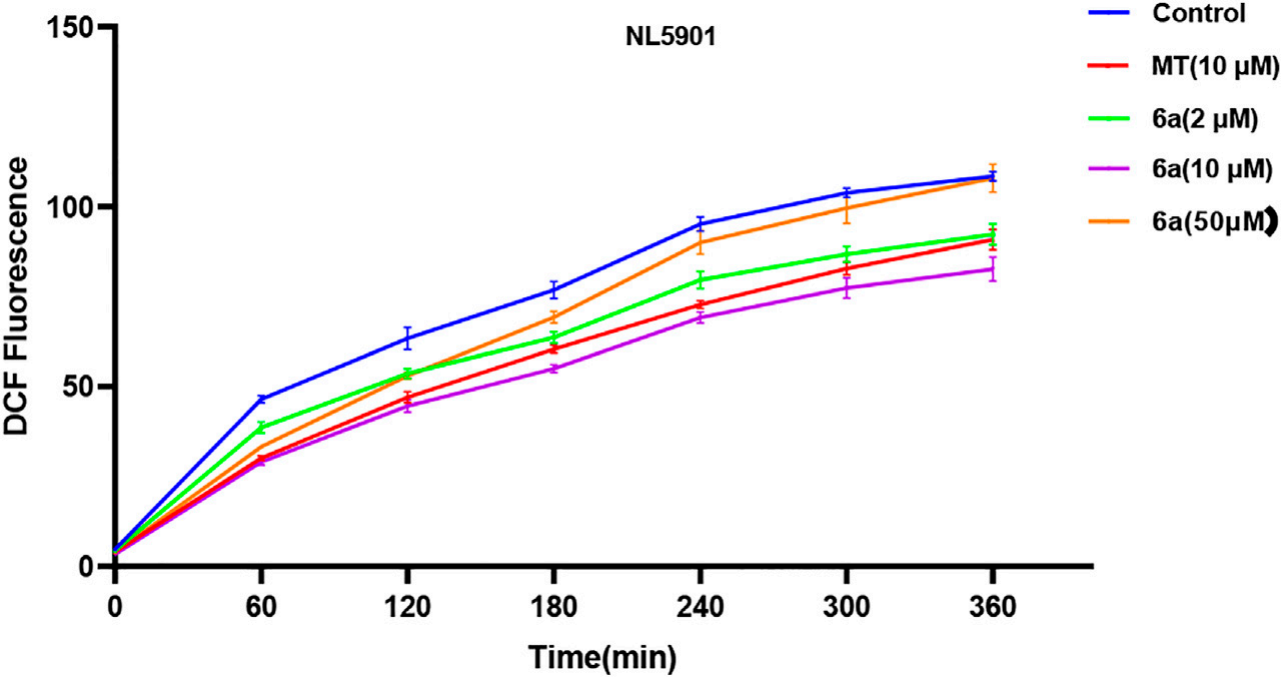
Effect of 6a on the ROS level of the transgenic nematode NL5901.

To test this hypothesis, we first examined the effect of 6a on the ROS level in the transgenic nematode model of PD. As shown in Figure 4, compared with the untreated group, after treatment with different concentrations (2, 10, and 50 µM) of 6a, the levels of ROS in NL5901 worms reduced, and 10 µM of 6a achieved a significant decrease compared with the level in the untreated group (*p* < 0.001), along with a clear decline compared with that in the MT group (*p* < 0.05). These results are consistent with the findings in the ROS levels in N2 worms. The regulation of ROS levels in wild-type and NL590 animals by **6a** confirmed its ability to regulate ROS *in vivo*, suggesting that it could reduce ROS-induced acute oxidative damage.

### 6a Reduced *α*-Synuclein Aggregation in the Transgenic Nematode NL5901

**FIGURE 5:**
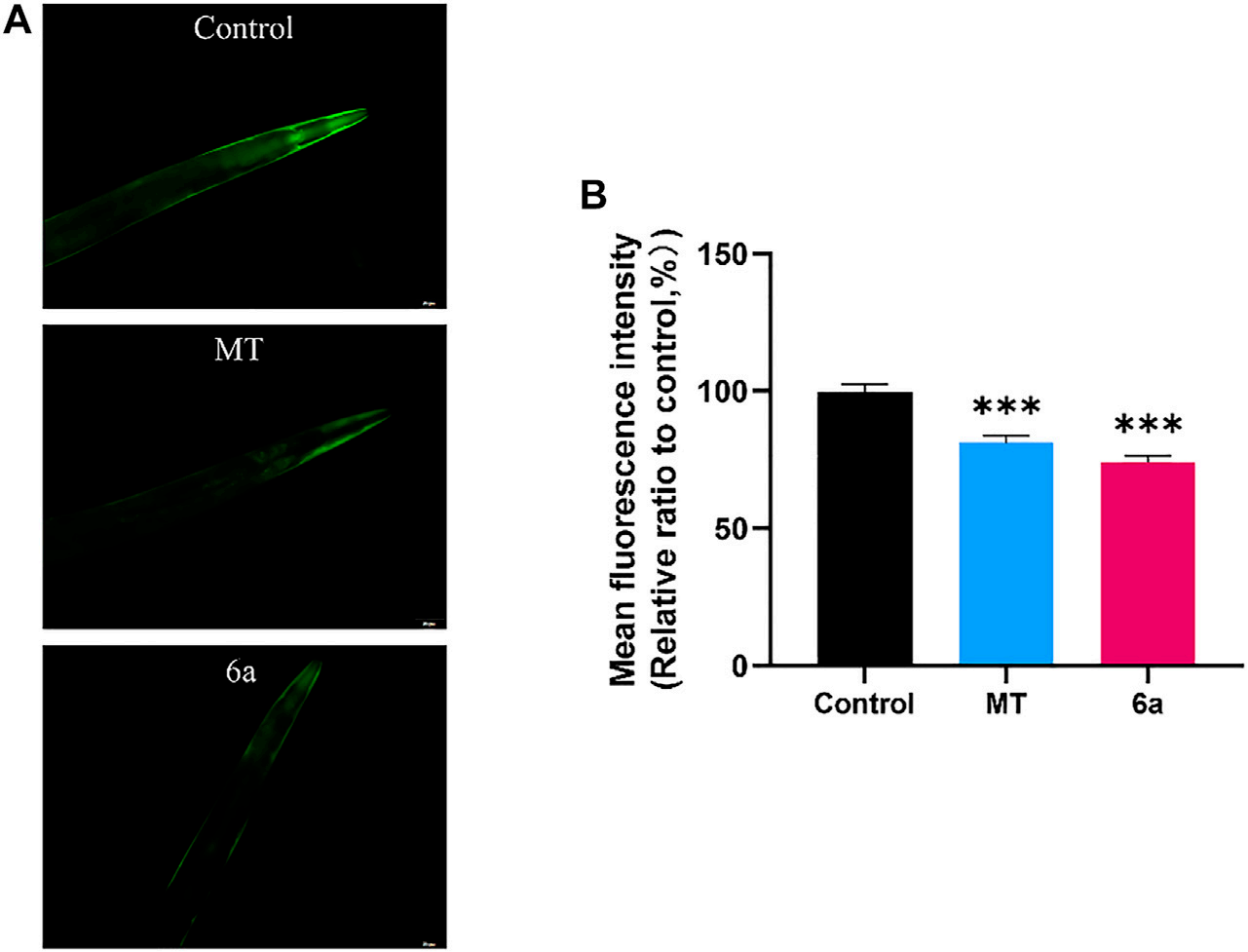
*α*-syn aggregation in NL5901 was decreased by treatment of MT and 6a. **(A)** Representative images of *α*-syn accumulation in different groups of head muscles. **(B)** Quantification of fluorescence intensity in NL5901 using ImageJ software (***p < 0.001).

One of the typical features of PD patients is the formation of Lewy bodies in the brain. The accumulation of Lewy bodies exacerbates the neurodegenerative progression in PD. Aggregated *α*-syn, which is known as a major component of Lewy bodies, can be measured in the transgenic NL5901 strain of *C. elegans*. In this strain, human *α*-syn is expressed in muscle cells with a YFP reporter. The fluorescence intensity at the anterior end of the worms indicates the accumulation of *α*-syn. To further confirm the therapeutic effect of 6a on PD, we examined its effect on *α*-syn aggregation. The results showed that, in the group treated with 10 µM 6a, the YFP fluorescence intensity in NL5901 was reduced compared with that in the untreated control group (Figure 5A). Quantitative analysis of the fluorescence intensity using ImageJ software showed that, in worms treated with 10 µM 6a (73.92 ± 1.80), the fluorescence intensity was lowered by 25.65% (*p* < 0.001) compared with that in untreated worms (99.45 ± 2.03). In addition, the 6a-treated group had lower fluorescence intensity than the MT-treated group (81.04 ± 2.74, *p* < 0.05; Figure 5B). These results indicate that the protective effect of 6a occurs by reducing the aggregation of *α*-syn in the PD model.

### 6a Is Protective Against 6-OHDA-Induced Dopaminergic Neuronal Damage

**FIGURE 6:**
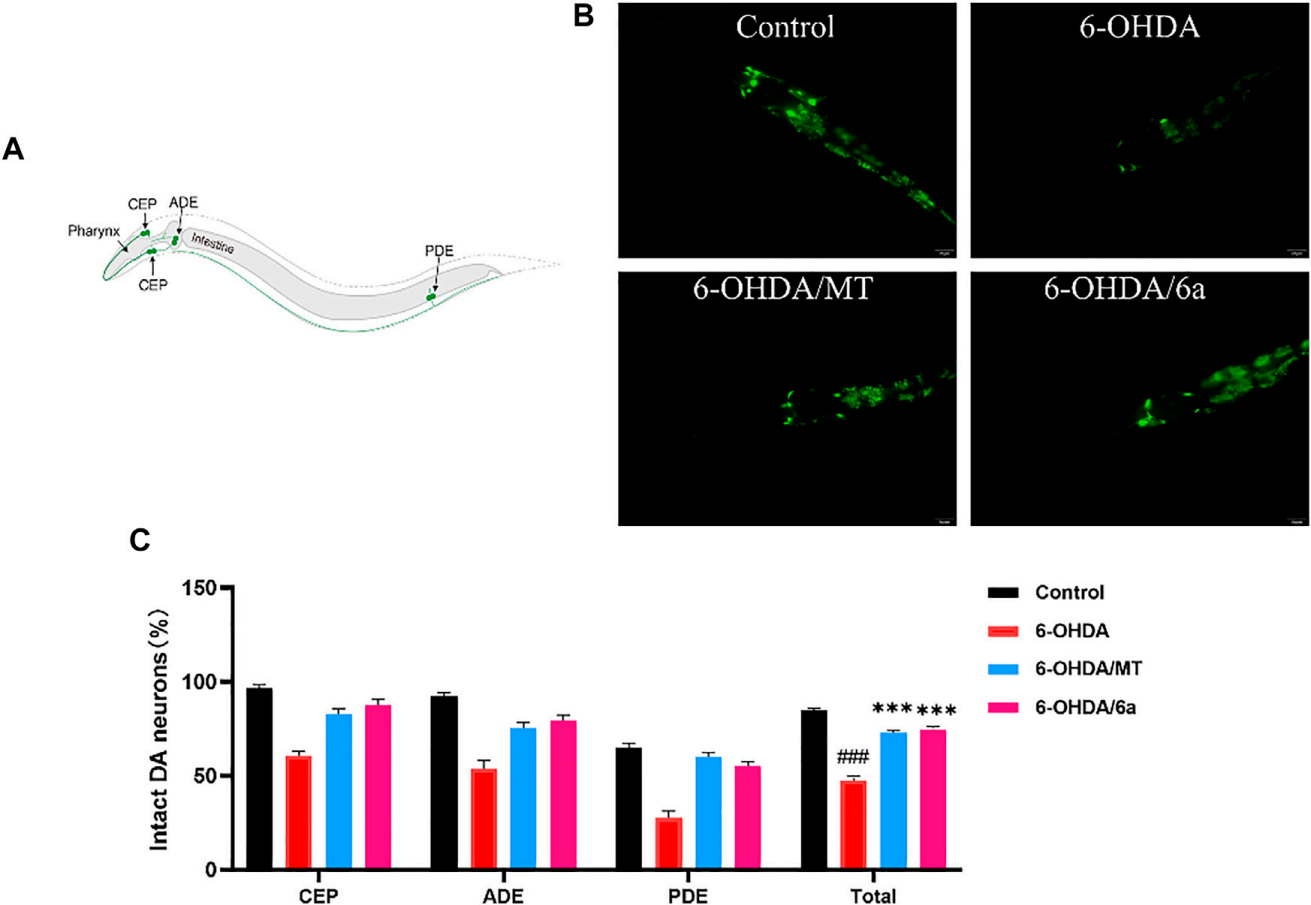
Effect of MT and 6a on restoration of 6-OHDA-induced DA neurodegeneration in the BZ555 strain. **(A)** Neuronal circuits in *C. elegans.* **(B)** GFP expression patterns of CEP and ADE in BZ555. **(C)** Quantification of the fluorescence intensity of all eight DA neurons of BZ555. (###*p* < 0.001, compared with the control; ***p < 0.001, compared with the 6-OHDA-treated group).

*C. elegans* contains exactly eight dopaminergic neurons, including two ADEs, four CEPs, and two PDEs (Figure 6A). In the BZ555 strain, all the eight DA neurons were tagged with GFP. Upon exposure to 50 mM 6-OHDA, the BZ555 strain showed selective damage in DA neurons. To examine the efficacy of 6a, the viability of the DA neurons was assessed by measuring the loss of expression of the GFP reporter. We found that CEP, ADE, and PDE neurons showed partial GFP loss after 6-OHDA treatment (Figure 6B). When nematodes were pretreated with MT or 6a for 48 h at L1, the GFP expression loss was remarkably restored in CEP and ADE neurons (Figure 6B). We further measured the fluorescence intensity in DA neurons using ImageJ software. In nematodes treated with 50 mM 6-OHDA (47.41 ± 2.53), the mean fluorescence (GFP) intensity decreased by about 56% (*p* < 0.001) compared with that of untreated nematodes (84.71 ± 1.25), whereas dopaminergic neurons of nematodes exposed to 6-OHDA after treatment with 10 µM 6a recovered to 74.28 ± 1.87 (*p* < 0.001), and the mean fluorescence intensity was higher in the 6a-treated group than in the MT-treated group (72.94 ± 1.09, *p* < 0.05; Figure 6C), suggesting that 6a is protective against 6-OHDA-induced dopaminergic neuronal damage and that this protective effect is stronger than that of MT.

### 6a Recovers Behavioral Dysfunction Induced by DA Neurodegeneration

**FIGURE 7:**
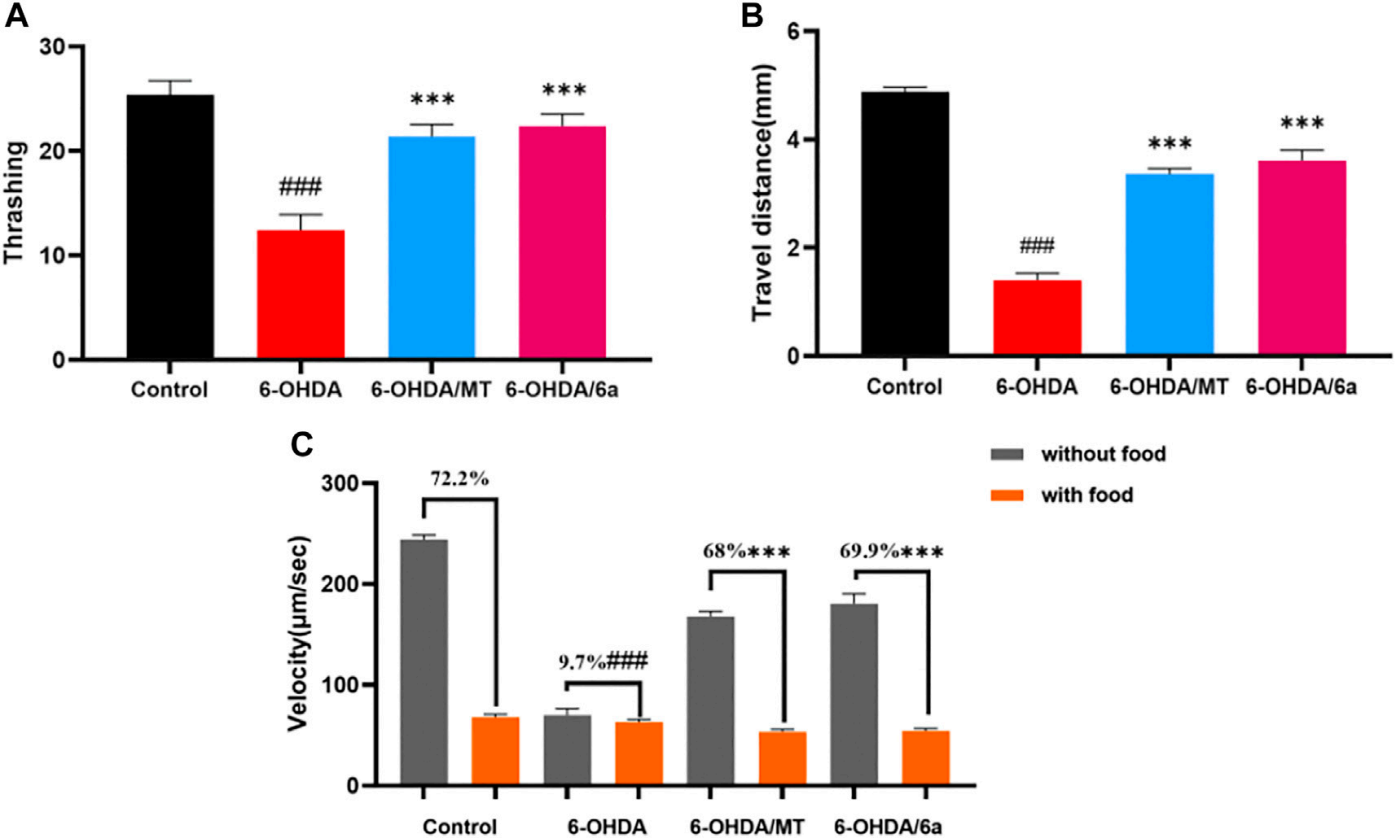
Effect of MT and 6a on DA neuronal function. **(A)** Thrashing behavior of N2. **(B)** Travel distances of N2. **(C)** Velocity of N2 in NGM plates with and without food and basal slowing response. (###*p* < 0.001, compared with the control; ***p < 0.001, compared with the 6-OHDA-treated group).

We next investigated whether 6a improves the functions of DA neurons. The locomotion of worms slows down when they encounter food, referred to as the basal slowing response, which is mediated by mechanosensory DA neurons. In this study, we found that N2 nematodes showed a 72.2% decrease in speed when on NGM plates with food and a 9.7% decrease in basal deceleration induced by 50 mM 6-OHDA (*p* < 0.001), in terms of food perception behavior. Meanwhile, there was significant recovery from this decline to 69.9% after treatment with 10 µM 6a (*p* < 0.001), and the 6a-treated group showed a better basal deceleration response than the melatonin-treated group (*p* < 0.05) (Figure 7C). Similarly, in wild-type N2 nematodes, supplementation with 6a also restored 6-OHDA-induced deficits in DA neuron-associated motor activity, such as increasing the distance traveled and thrashing behavior (Figures 7A, B). These results suggest that 6a recovers the behavioral dysfunction induced by DA neurodegeneration.

## Conclusion

We successfully synthesized 10 melatonin derivatives 6a–6j, and their activities were tested using *in vitro* antioxidant assays, ABTS and ORAC. The results showed that the antioxidant activity of melatonin derivative 6a was higher than that of MT. Subsequently, we evaluated the effect of 6a on ROS levels in *C. elegans*. The results showed that 10 µM 6a significantly reduced the ROS levels in wild-type N2. We also used two PD models of *C. elegans* to investigate the therapeutic effect of 6a on PD*.* We found that 10 µM 6a significantly reduced the ROS levels and *α*-syn aggregation in NL5901. Treatment of BZ555 with 50 mM 6-OHDA reduced the fluorescence intensity of their dopaminergic neuronal cells, which was increased by supplementation with 10 µM 6a. Finally, we evaluated the capacity of 6a to improve behavioral deficits caused by DA neurodegeneration. The results showed that 10 µM 6a improved the nematode’s behavior in perceiving food and increased its basal rate compared with the findings in the control.

Our conclusions prove that melatonin derivative 6a significantly reduces *α*-syn aggregation and dopaminergic neuronal damage in PD by reducing oxidative stress-induced ROS levels and improves the behavioral impairment caused by DA neurodegeneration. Further study of the mechanism of action of this compound could provide new therapeutic ideas and treatment strategies for PD.

## Data Availability

The original contributions presented in the study are included in the article/Supplementary Material; further inquiries can be directed to the corresponding authors.
