# Supplementary material for: Synthesis of Melatonin Derivatives and the Neuroprotective Effects on Parkinson’s Disease Models of Caenorhabditis elegans
Source: Front Chem. 2022 Jun 8;10:918116. doi: 10.3389/fchem.2022.918116 (PMC9213837; doi:10.3389/fchem.2022.918116)
Supplement: Supplementary file 1 [file DataSheet1.PDF]

Supporting Information

CONTENTS

1. **General Procedure for Synthesis of *N*-P*t*Bu2 Protected Melatonin**................1
2. **General Procedure for the Synthesis of C-7 Styryl Melatonin**........................1
3. **Experimental Procedure for the Synthesis of Compound 6** 2
4. **Spectroscopic data** 7
5. **Mass Spectrometry Data**...21

1. General Procedure for Synthesis of *N*-P*t*Bu2 Protected Melatonin

To a solution of melatonin (**1**) (8.0 mmol, 1.0 equiv) in 20 mL anhydrous THF at 0 °C was added a solution of *n*-BuLi (solution in hexanes, 20.0 mmol, 2.5 equiv) dropwise. After stirring for 15 min, di-tert-butylchlorophosphine (**2**) (12.0 mmol, 1.5 equiv) was added dropwise. The mixture was allowed to stir and warm to room temperature over several hours. After melatonin was consumed determined by TLC, the reaction was quenched by 4 mL MeOH. Then the solvent was removed under reduced pressure. Further purificationthrough flash chromatography(PE : EA = 1 : 1) to get the pure product, white solid (**3**).

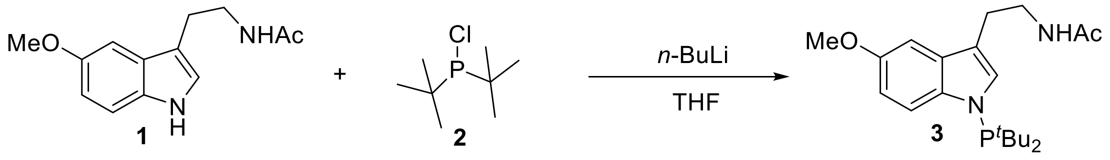

N-(2-(1-(di-tert-butylphosphaneyl)-5-methoxy-1H-indol-3-yl)ethyl)acetamide(3)

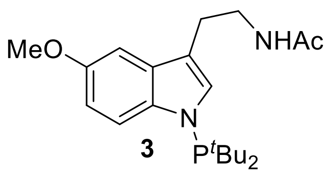

White solid. 90% yield. m.p.128～131 °C; **^{1}H NMR (500 MHz, Chloroform-*d*)** *δ*: 7.76 (dd, *J* =8.9 Hz, 2.4 Hz, 1H), 7.26 (s, 1H), 6.99 (dd, *J* =2.6 Hz, 1.1 Hz, 1H), 6.89 (dd, *J* =8.9 Hz, 2.5 Hz, 1H), 5.77 (d, *J* = 5.8 Hz, 1H), 3.87 (s, 3H), 3.61 (q, *J* = 6.5 Hz, 2H), 3.01～2.94 (m, 2H), 1.94 (s, 3H), 1.22 (d, *J* =12.8 Hz, 18H) ; **^{13}C NMR (125 MHz, Chloroform-*d*)** δ 170.0, 154.3, 139.4, 129.1, 128.5, 115.2, 113.9, 111.8, 100.2, 55.8, 39.4, 35.2, 35.0, 29.1, 25.4, 23.3 ; LC-MS (ESI) *m/z*: calcd. for $C_{21}H_{33}N_2O_2P$ {$[M+Na]^+$} 399.2172, found 399.2171。

2. General Procedure for the Synthesis of C-7 Styryl Melatonin

To a 25 mL Schlenk tube was added **3** (0.2 mmol), substituted cinnamic acid **4** (0.5 mmol), $Rh(cod)_2OTf$ (4.7 mg, 0.01 mmol) and Boc_2O (109 mg, 0.5 mmol), the tube was purged with Ar three times, followed by addition of anhydrous toluene (2 mL) The formed mixture was stirred at 120 °C under Ar for 18 h as monitored by TLC. The solution was then cooled to room temperature. The solvent was removed under vaccum

directly. The crude product was purified by flash column chromatography on silica gel affording pure products **5**.

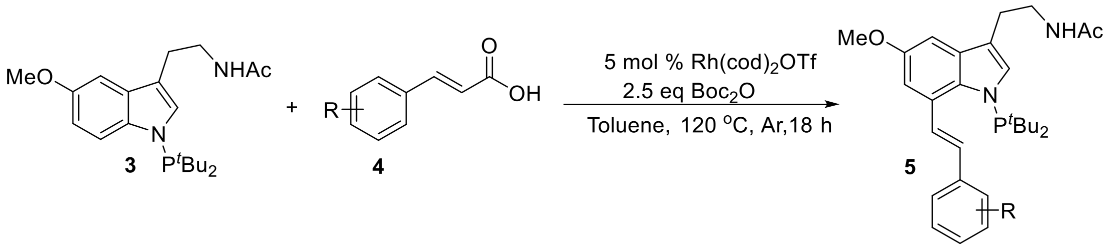

N-(2-(1-(di-tert-butylphosphaneyl)-5-methoxy-7-styryl-1H-indol-3-yl)ethyl)acetamide(5a)

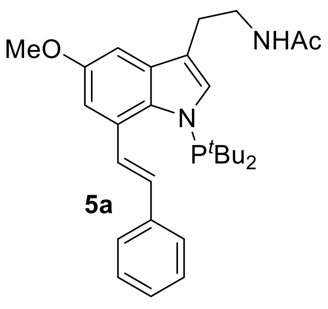

Light yellow oil. 65% yield. **^{1}H NMR** (**500 MHz, Chloroform-*d***) δ: 8.64 (dd, J =16.0 Hz, 2.3 Hz, 1H), 7.59～7.54 (m, 2H), 7.37 (t, J =7.7 Hz, 2H), 7.04 (d, J =2.6 Hz, 1H), 6.93 (dd, J =2.6 Hz, 1.3 Hz, 2H), 6.77 (dd, J =16.0 Hz, 1.4 Hz, 2H), 5.47 (s, 1H), 3.88 (s, 3H), 3.60 (q, J =6.5 Hz, 2H), 2.97～2.91 (m, 2H), 1.91 (s, 3H), 1.20 (d, J =12.7 Hz, 18H); **^{13}C NMR** (**125 MHz, Chloroform-*d***) δ: 170.0, 154.3, 138.4, 135.9, 130.8, 130.0, 129.7, 129.5, 128.7, 128.0, 127.1, 126.7, 126.5, 115.3, 111.3, 100.0, 55.8, 39.3, 36.3, 36.0, 29.4, 25.3, 23.4; **^{31}P NMR** (**202 MHz, Chloroform-*d***) δ: 71.5.

3. Experimental Procedure for the Synthesis of Compound 6

To the above product **5** (0.1 mmol) in 25 mL Schlenk tube was added anhydrous THF (1.2 mL), and then TBAF (0.8 mL, 0.8 mmol, 1 M in THF) was added, the mixture was stirred under 60 ^{o}C until raw materials was consumed as determined by TLC. The reaction was cooled to room temperature and 10 mL H_2O was added, then the mixture was extracted by ethyl acetate and the organic pahse was combined and dried with anhydrous Na_2SO_4. The solvent was evaporated under reduced pressure and the crude was purified by column chromatography on silica gel to provide **6** as light yellow oil.

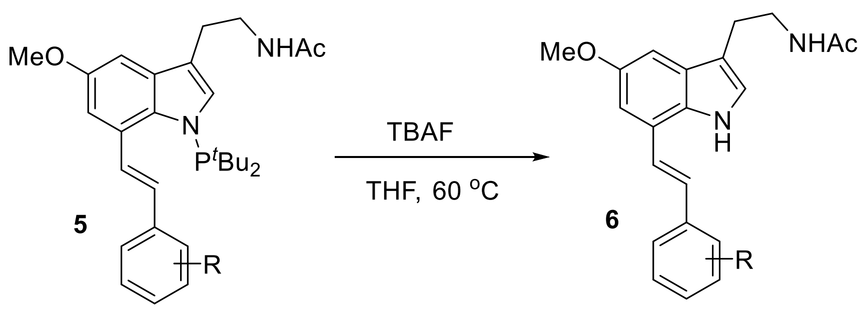

N-(2-(5-methoxy-7-styryl-1H-indol-3-yl)ethyl)acetamide(6a)

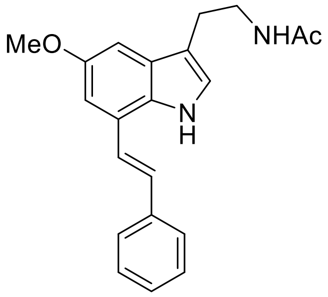

6a

Light yellow oil. 67% yield. **^{1}H NMR** (**500 MHz, Chloroform-*d***) *δ*: 8.47 (s, 1H), 7.60～7.49 (m, 2H), 7.44～7.35 (m, 2H), 7.35～7.27 (m, 2H), 7.18 (s, 1H), 7.09 (t, *J* =2.0 Hz, 2H), 7.03 (d, *J* =2.3 Hz, 1H), 5.62 (s, 1H), 3.92 (s, 3H), 3.62 (q, *J* =6.5 Hz, 2H), 2.97 (t, *J* =6.8 Hz, 2H), 1.94 (s, 3H); **^{13}C NMR** (**125 MHz, Chloroform-*d***) *δ*: 170.2, 154.4, 137.2, 130.5, 129.8, 128.8, 128.5, 127.9, 126.5, 124.3, 122.9, 122.3, 113.2, 109.9, 100.7, 56.1, 39.8, 25.3, 23.4; LC-MS (ESI) *m/z*: calcd. for $C_{21}H_{22}N_2O_2$ {[M+Na]$^+$} 357.1573, found 357.1572。

N-(2-(5-methoxy-7-(4-methylstyryl)-1H-indol-3-yl)ethyl)acetamide(6b)

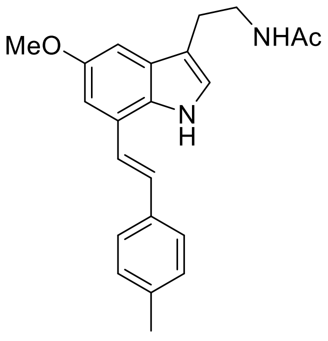

6b

Light yellow oil. 47% yield. **^{1}H NMR** (**500 MHz, Chloroform-*d***) *δ*: 8.33 (s, 1H), 7.47 (d, *J* =8.0 Hz, 2H), 7.30～7.26 (m, 1H), 7.22 (d, *J* =7.9 Hz, 2H), 7.17 (d, *J* =16.3 Hz, 1H), 7.08 (dd, *J* = 12.6 Hz, 2.4 Hz, 2H), 7.02 (d, *J* =2.3 Hz, 1H), 5.58 (s, 1H), 3.92 (s, 3H), 3.63 (q, *J* =6.5 Hz, 2H), 2.98 (t, *J* =6.7 Hz, 2H), 2.40 (s, 3H), 1.96 (s, 3H); **^{13}C NMR** (**125 MHz, Chloroform-*d***) *δ*: 170.1, 154.4, 137.9, 134.4, 130.5, 129.8, 129.5, 128.5, 126.4, 123.3, 122.8, 122.5, 113.2, 109.9, 100.5, 56.1, 39.7, 25.3, 23.5, 21.3; LC-MS (ESI) *m/z*: calcd. for $C_{22}H_{24}N_2O_2${[M+Na]$^+$} 371.1730, found 371.1728。

N-(2-(7-(2-chlorostyryl)-5-methoxy-1H-indol-3-yl)ethyl)acetamide（6c）

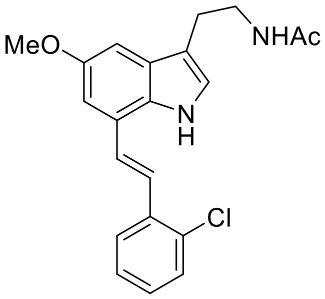

6c

Light yellow oil. 50% yield. **^{1}H NMR** (**500 MHz, Chloroform-*d***) *δ*: 8.57 (s, 1H), 7.39～7.32 (m, 2H), 7.31 (d, *J* =8.3 Hz, 2H), 7.15 (d, *J* =16.3 Hz, 1H), 7.08 (dd, *J* =3.9 Hz, 2.3 Hz, 2H), 7.04 (d, *J* =2.3 Hz, 1H), 7.00 (m, 1H), 5.63 (s, 1H), 3.91 (s, 3H), 3.62 (q, *J* =6.6 Hz, 2H), 2.97 (t, *J* =6.8 Hz, 2H), 1.97 (s, 3H); **^{13}C NMR** (125 MHz, Chloroform-*d*) *δ*: 170.2, 154.4, 133.4, 129.7, 129.3, 128.5, 128.0, 128.0, 124.1, 124.1, 122.9, 122.2, 115.9, 115.7, 113.3, 110.0, 100.7, 56.1, 39.8, 25.3, 23.4; LC-MS (ESI) *m/z*: calcd. for $C_{21}H_{21}ClN_2O_2$ {[M+Na]$^{+}$} 391.1184, found 391.1183。

N-(2-(5-methoxy-7-(2-methoxystyryl)-1H-indol-3-yl)ethyl)acetamide(6d)

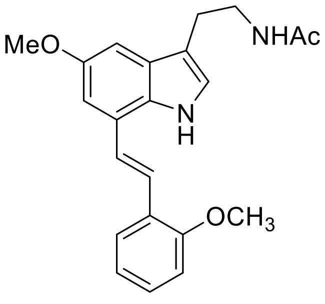

6d

Light yellow oil. 85% yield. **^{1}H NMR** (**500 MHz, Chloroform-*d***) *δ*: 8.40～8.36 (m, 1H), 7.66 (dd, *J* =7.7 Hz, 1.8 Hz, 1H), 7.55 (d, *J* =16.5 Hz, 1H), 7.34 (s, 1H), 7.30 (d, *J* =2.4 Hz, 1H), 7.09 (dd, *J* =7.3 Hz, 2.4 Hz, 2H), 7.04～7.00 (m, 2H), 6.95 (dd, *J* =8.4 Hz, 1.1 Hz, 1H), 5.58 (s, 1H), 3.92 (d, *J* =3.8 Hz, 6H), 3.63 (q, *J* =6.5 Hz, 2H), 2.98 (t, *J* =6.7 Hz, 2H), 1.96 (s, 3H); **^{13}C NMR** (**125 MHz, Chloroform-*d***) *δ*: 170.1, 156.9, 154.4, 129.7, 129.0, 128.4, 126.4, 126.3, 125.4, 125.0, 123.0, 122.8, 120.8, 113.1, 111.0, 110.4, 100.4, 56.1, 55.6, 39.7, 25.3, 23.5; LC-MS (ESI) *m/z*: calcd. for $C_{22}H_{24}N_2O_3$ {[M+Na]$^{+}$} 387.1679, found 387.1677。

N-(2-(7-(4-chlorostyryl)-5-methoxy-1H-indol-3-yl)ethyl)acetamide(6e)

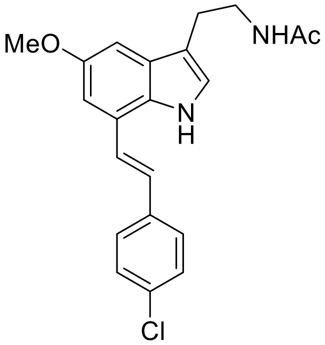

6e

Light yellow oil. 52% yield. **^{1}H NMR** (**500 MHz, Chloroform-*d***) *δ*: 8.46 (s, 1H),7.57～ 7.50 (m, 2H), 7.25 (s, 1H), 7.15 (d, *J* =16.2 Hz, 1H), 7.12～7.05 (m, 4H), 7.03 (d, *J* =2.3 Hz, 1H), 5.62 (s, 1H).3.91 (s, 3H), 3.62 (q, *J* =6.5 Hz, 2H), 2.97 (t, *J* =6.8 Hz, 2H), 1.96 (s, 3H); **^{13}C NMR** (**125 MHz, Chloroform-*d***) *δ*: 170.2, 154.5, 134.9, 134.8, 134.5, 131.1, 130.3, 129.4, 127.9, 127.7, 126.1, 113.3,

111.9, 110.5, 100.6, 55.9, 40.0, 24.6, 23.3; LC-MS (ESI) *m/z*: calcd. for $C_{21}H_{21}ClN_2O_2$ {[M+Na]$^+$} 391.1184, found 391.1181。

N-(2-(7-(3-fluorostyryl)-5-methoxy-1H-indol-3-yl)ethyl)acetamide(6f)

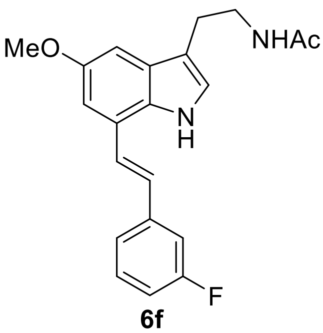

6f

Light yellow oil. 54% yield. **^{1}H NMR** (**500 MHz, Chloroform-*d***) δ: 8.57 (s, 1H), 7.39～7.33 (m, 2H), 7.31 (dd, *J* =8.9 Hz, 2.0 Hz, 2H), 7.15 (d, *J* =16.2 Hz, 1H), 7.08 (dd, *J* =4.0 Hz, 2.3 Hz, 2H), 7.04 (d, *J* =2.3 Hz, 1H), 7.02～6.98 (m, 1H), 5.63 (s, 1H), 3.91 (s, 3H), 3.62 (q, *J* =6.6 Hz, 2H), 2.97 (t, *J* =6.8 Hz, 2H), 1.97 (s, 3H); **^{13}C NMR** (**125 MHz, Chloroform-*d***) δ: 170.2, 163.2, 154.3, 139.6, 130.2, 129.9, 129.1, 128.6, 125.6, 123.0, 122.6, 121.8, 114.6, 113.2, 112.7, 109.9, 101.1, 56.1, 39.8, 25.3, 23.4; **^{19}F NMR** (**471 MHz, Chloroform-*d***) δ: -113.7; LC-MS (ESI) *m/z*: calcd. for $C_{21}H_{21}FN_2O_2$ {[M+Na]$^+$} 375.1479, found 375.1476。

N-(2-(5-methoxy-7-(2-methylstyryl)-1H-indol-3-yl)ethyl)acetamide(6g)

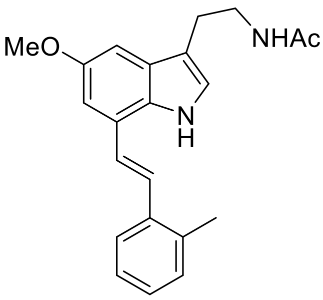

6g

Light yellow oil. 67% yield. **^{1}H NMR** (**500 MHz**, **Chloroform-*d***) δ: 8.30 (s, 1H), 7.68～7.63 (m, 1H), 7.42 (d, *J* =16.1 Hz, 1H), 7.28～7.25 (m, 1H), 7.25～7.22 (m, 2H), 7.20 (s, 1H), 7.10 (d, *J* =2.3 Hz, 2H), 7.04 (d, *J* =2.3 Hz, 1H), 5.58 (s, 1H), 3.93 (s, 3H), 3.63 (q, *J* =6.5 Hz, 2H), 2.98 (t, *J* =6.7 Hz, 2H), 2.46 (s, 3H), 1.96 (s, 3H); **^{13}C NMR** (**125 MHz**, **Chloroform-*d***) δ: 170.1, 154.4, 136.3, 135.9, 130.5, 129.8, 128.6, 128.5, 127.9, 126.3, 125.4, 125.4, 122.9, 122.7, 113.3, 110.0, 100.4, 56.1, 39.8, 25.3, 23.5, 20.0; LC-MS (ESI) *m/z*: calcd. for $C_{22}H_{24}N_2O_2$ {[M+Na]$^+$} 371.1730, found 371.1728。

N-(2-(5-methoxy-7-(4-methoxystyryl)-1H-indol-3-yl)ethyl)acetamide(6h)

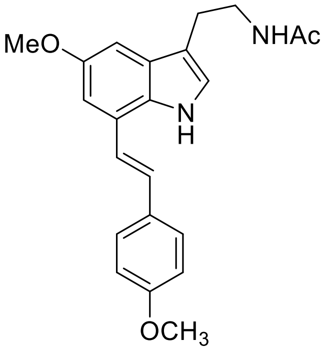

6h

Light yellow oil. 59% yield. **^{1}H NMR** (**500 MHz**, **Chloroform-*d***) δ: 8.47 (s, 1H), 7.52～7.48 (m, 1H), 7.30～7.27 (m, 1H), 7.24～7.12 (m, 2H), 7.05 (m, 2H), 7.00 (d, *J* =2.3 Hz, 1H), 6.96～6.92 (m, 1H), 6.89 (dd, *J* =8.7 Hz, 2.4 Hz, 1H), 5.62 (s, 1H), 3.93～3.84 (m, 6H), 3.61 (m, 2H), 2.96 (td, *J* =6.7 Hz, 4.4 Hz, 2H), 1.95 (d, *J* =3.0 Hz, 3H); **^{13}C NMR** (**125 MHz**, **Chloroform-*d***) δ: 170.2, 159.5, 154.4, 130.1, 129.8, 128.4, 127.8, 122.8, 122.1, 114.2, 112.5, 112.0, 109.7, 100.4, 100.2, 56.1, 55.4, 39.8, 25.3, 23.4; LC-MS (ESI) *m/z*: calcd. for $C_{22}H_{24}N_2O_3$ {$[M+Na]^+$} 387.1679, found 387.1675。

N-(2-(7-(3,4-dimethoxystyryl)-5-methoxy-1H-indol-3-yl)ethyl)acetamide(6i)

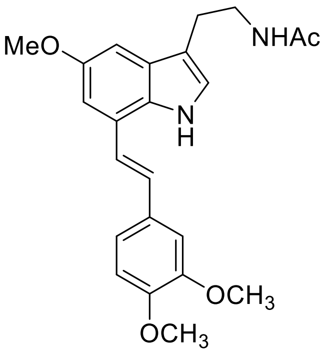

6i

Light yellow oil. 49% yield. **^{1}H NMR** (**500 MHz**, **Chloroform-*d***) δ: 8.47 (s, 1H), 7.24～7.14 (m, 2H), 7.11 (d, *J* =7.9 Hz, 2H), 7.08 (d, *J* =2.3 Hz, 1H), 7.06 (d, *J* =2.4 Hz, 1H), 7.01 (d, *J* =2.3 Hz, 1H), 6.90 (d, *J* =8.0 Hz, 1H), 5.62 (s, 1H), 3.99～3.90 (m, 9H), 3.61 (p, *J* =6.1 Hz, 5.7 Hz, 2H), 2.97 (t, *J* =6.7 Hz, 2H), 1.96 (s, 3H); **^{13}C NMR** (**125 MHz, Chloroform-*d***) δ: 170.1, 154.4, 149.2, 149.2, 130.4, 130.3, 129.8, 128.5, 122.8, 122.6, 122.5, 119.9, 113.2, 111.3, 109.8, 108.8, 100.3, 56.1, 56.0, 56.0, 39.7, 25.3, 23.4; LC-MS (ESI) *m/z*: calcd. for $C_{23}H_{26}N_2O_4$ {$[M+Na]^+$} 417.1785, found 417.1781。

N-(2-(7-(4-fluorostyryl)-5-methoxy-1H-indol-3-yl)ethyl)acetamide(6j)

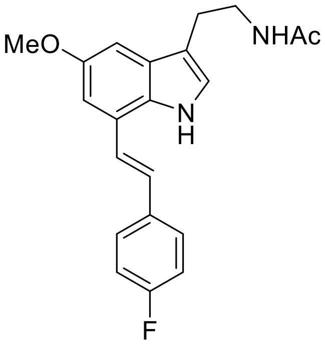

6j

Light yellow oil. 56% yield. **^{1}H NMR (500 MHz, Chloroform-*d*)** δ: 8.39 (d, J =13.1 Hz, 1H), 7.57～7.52 (m, 2H), 7.24 (s, 1H), 7.15 (d, J =16.3 Hz, 1H), 7.12～7.07 (m, 3H), 7.06 (d, J =2.3 Hz, 1H), 7.03 (t, J =1.6 Hz, 1H), 5.63 (d, J =29.4 Hz, 1H), 3.91 (s, 3H), 3.62 (q, J =6.5 Hz, 2H), 2.97 (t, J =6.7 Hz, 2H), 1.96 (s, 3H); **^{13}C NMR (125 MHz, Chloroform-*d*)** δ: 170.2, 162.5, 154.4, 133.4, 129.8, 129.2,128.5, 128.0, 124.1, 122.9, 122.2, 115.7, 113.2, 109.8, 100.7, 56.1, 39.8, 25.3, 23.4; ^{19}F NMR (471 MHz, Chloroform-*d*) δ: -113.2; LC-MS (ESI) *m/z*: calcd. for $C_{21}H_{21}FN_2O_2$ {[M+Na]$^{+}$} 375.1479, found 375.1481。

4. Spectroscopic data

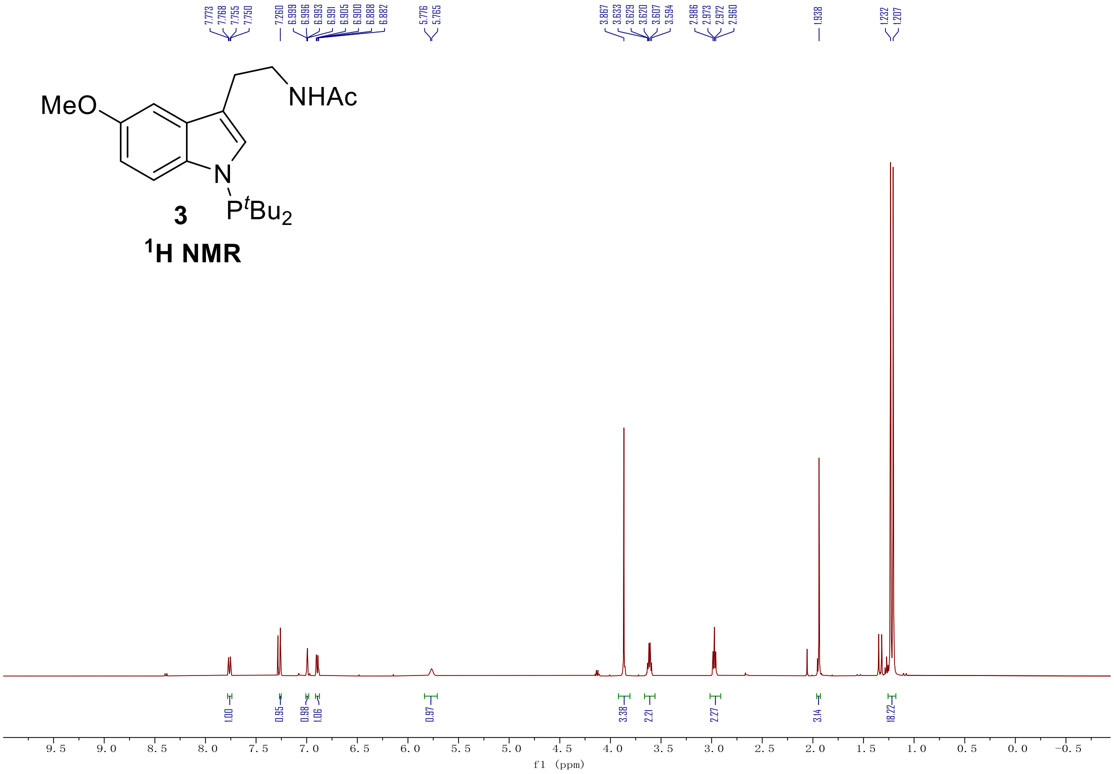

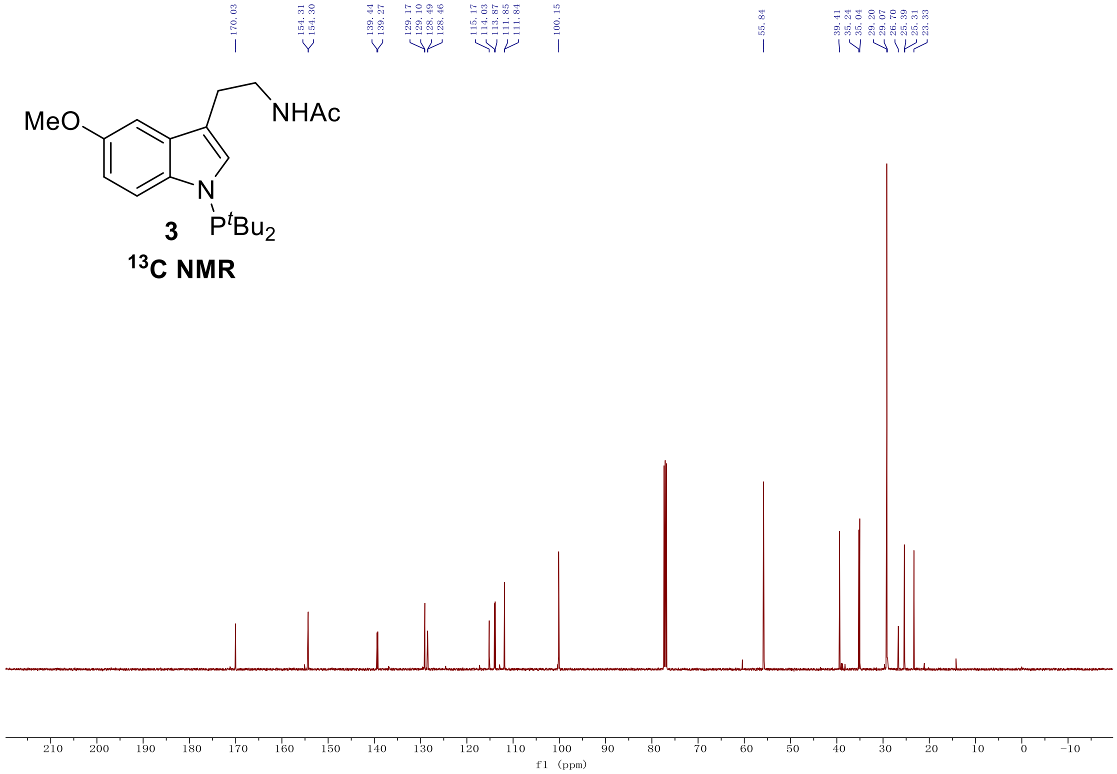

170.03
154.31
154.30
139.44
139.27
129.17
129.10
128.49
128.46
115.17
114.03
113.87
111.85
111.84
100.15
55.84
39.41
35.24
35.04
29.20
29.07
26.70
25.39
23.31
23.33
MeO
NHAc
N
P^{t}Bu$_2$
3
^{13}C NMR
f1 (ppm)

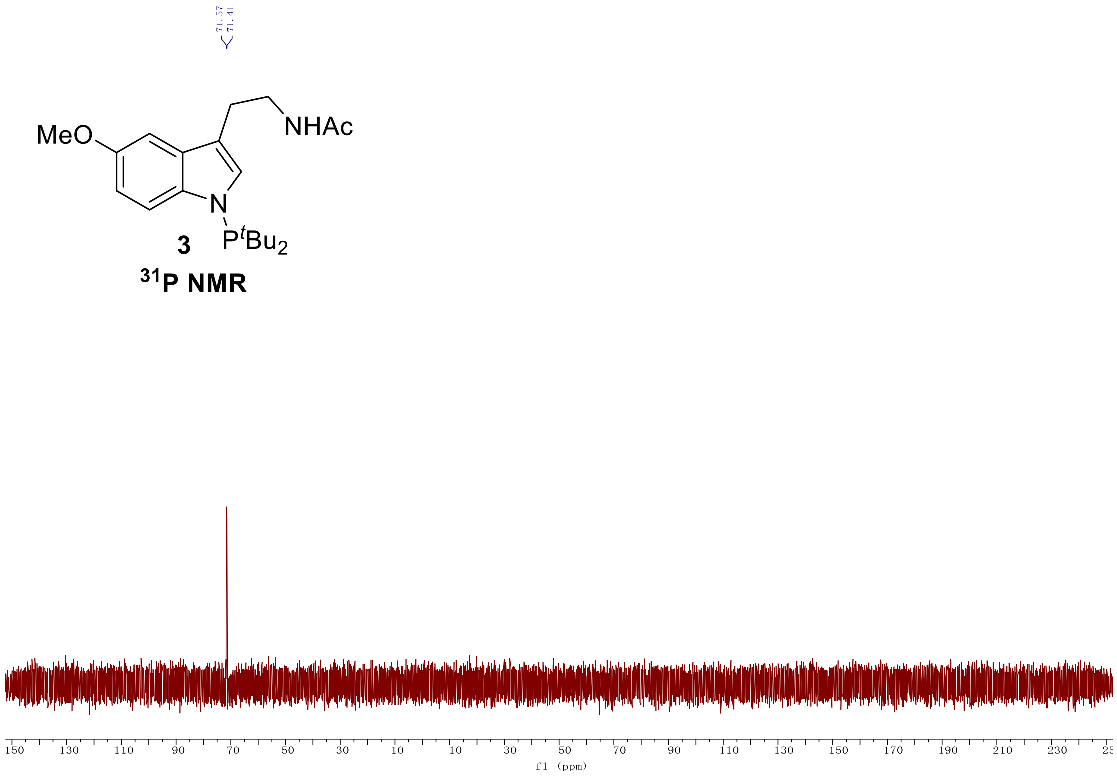

71.57
71.41
MeO
NHAc
N
P^{t}Bu$_2$
3
^{31}P NMR
f1 (ppm)

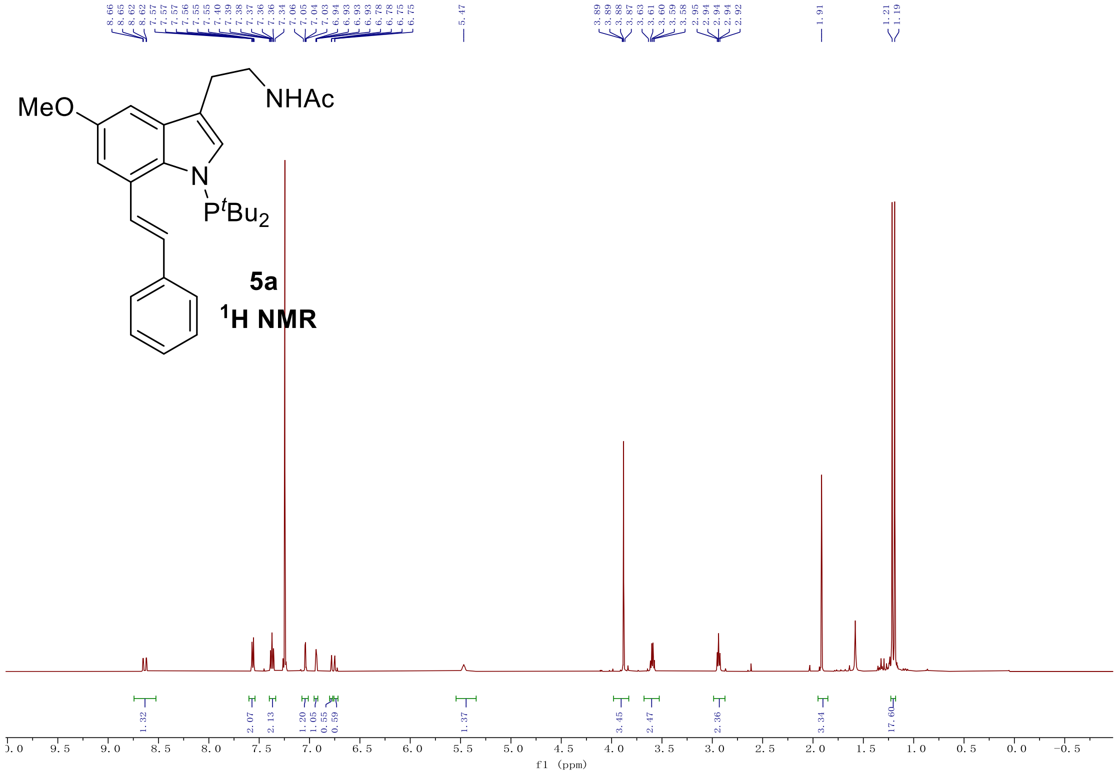

8.66 8.65 8.62 8.62 7.57 7.57 7.56 7.55 7.55 7.40 7.39 7.38 7.37 7.36 7.36 7.34 7.06 7.05 7.04 7.03 6.94 6.93 6.93 6.93 6.78 6.78 6.75 6.75
5.47
3.89 3.89 3.88 3.87 3.63 3.61 3.60 3.59 3.58 2.95 2.94 2.94 2.94 2.92
1.91
1.21 1.19
MeO
NHAc
N
P^tBu2
5a
1H NMR
1.32 2.07 2.13 1.20 1.05 0.55 0.59 1.37 3.45 2.47 2.36 3.34 17.60
f1 (ppm)

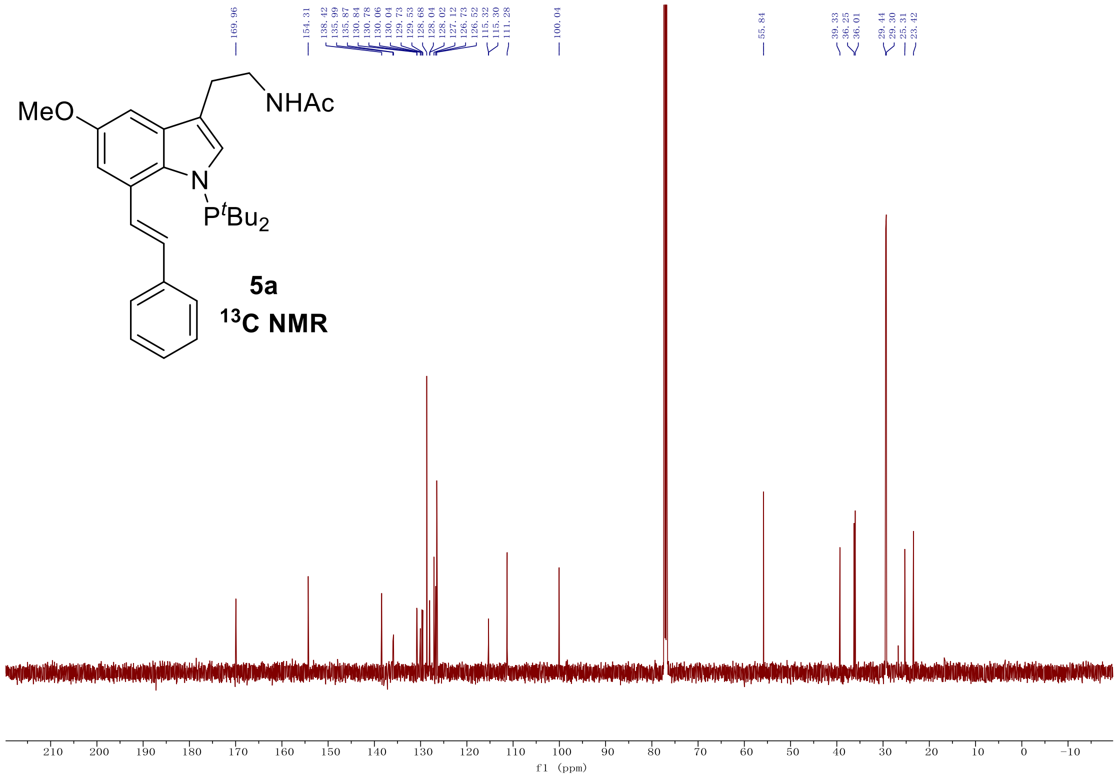

169.96
154.31
138.42 135.99 135.87 130.84 130.78 130.06 130.04 129.73 129.53 128.68 128.04 128.02 127.12 126.73 126.52 115.32 115.30 111.28
100.04
55.84
39.33 36.25 36.01
29.44 29.30 25.31 23.42
MeO
NHAc
N
P^tBu2
5a
13C NMR
f1 (ppm)

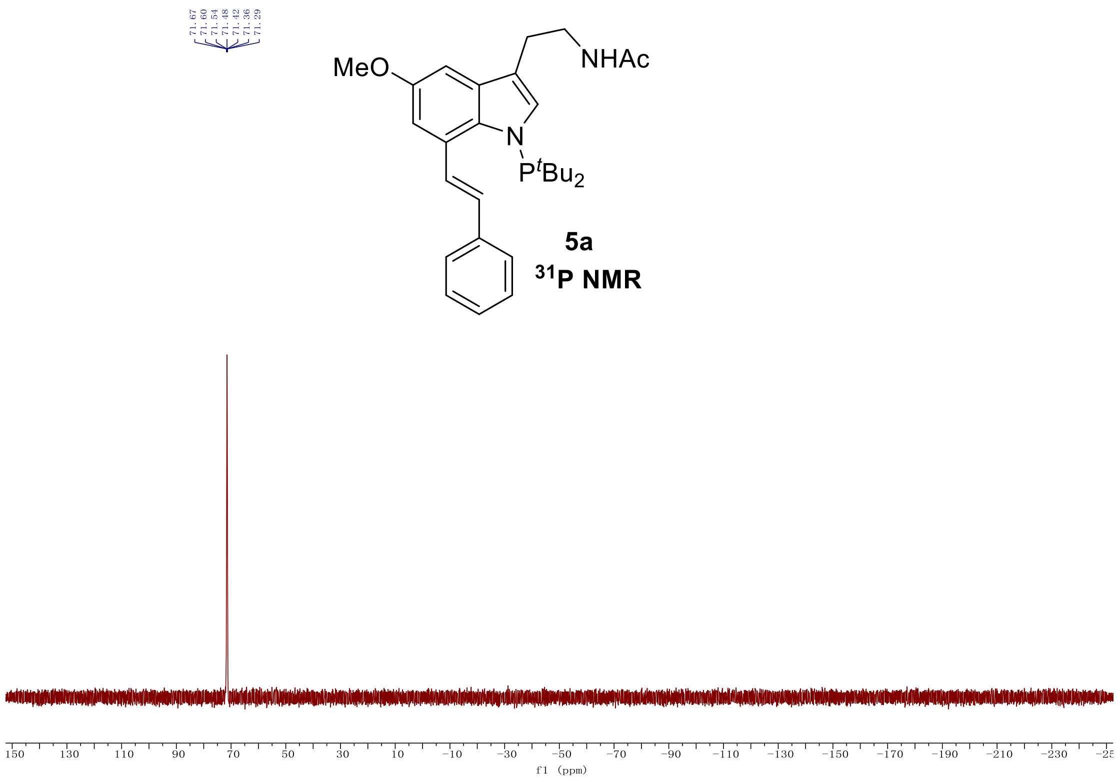
MeO
NHAc
N
P^{t}Bu$_2$
5a
^{31}P NMR
f1 (ppm)

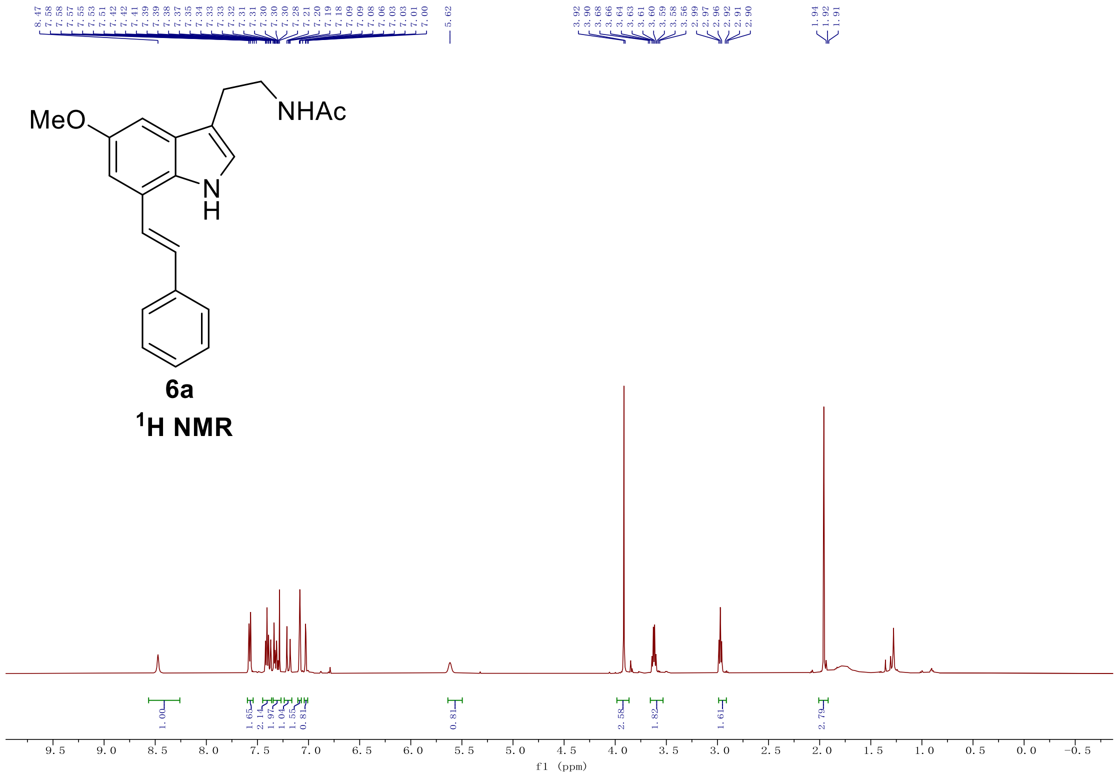
MeO
NHAc
N
H
6a
^{1}H NMR
f1 (ppm)

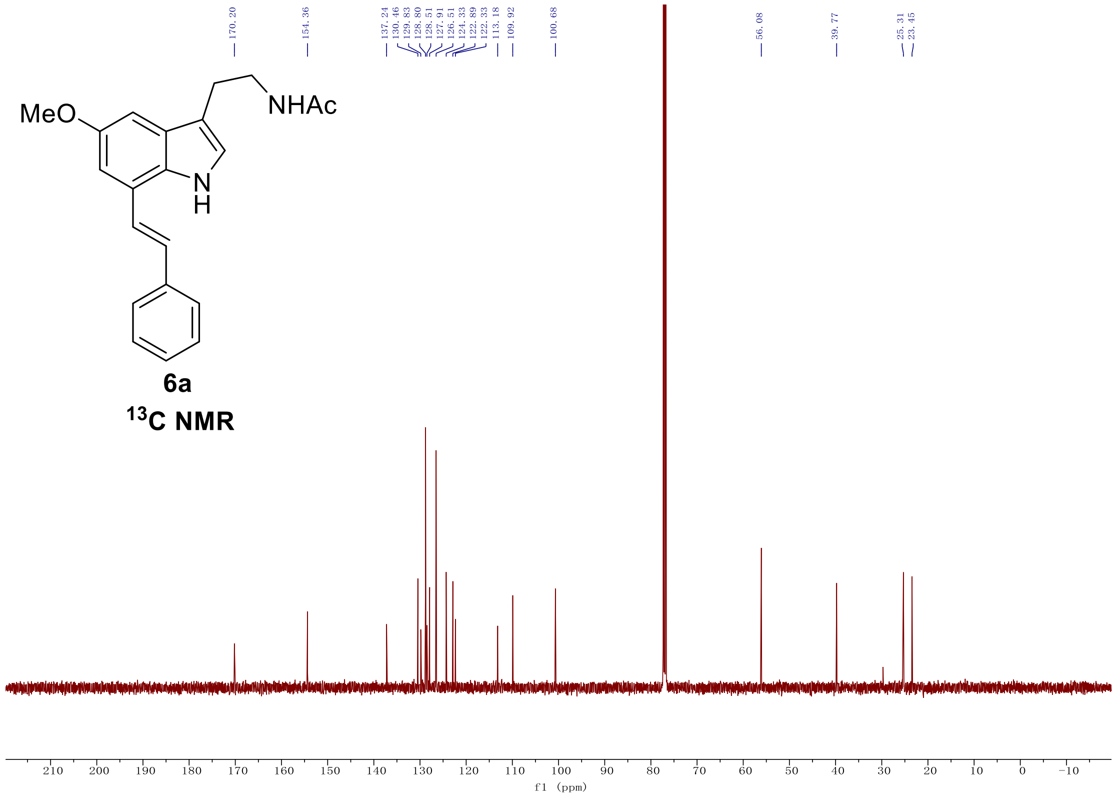
170.20
154.36
137.24
130.46
129.83
128.80
128.51
127.91
126.51
124.33
122.89
122.33
113.18
109.92
100.68
56.08
39.77
25.31
23.45
MeO
NHAc
N
H
6a
13C NMR
f1 (ppm)

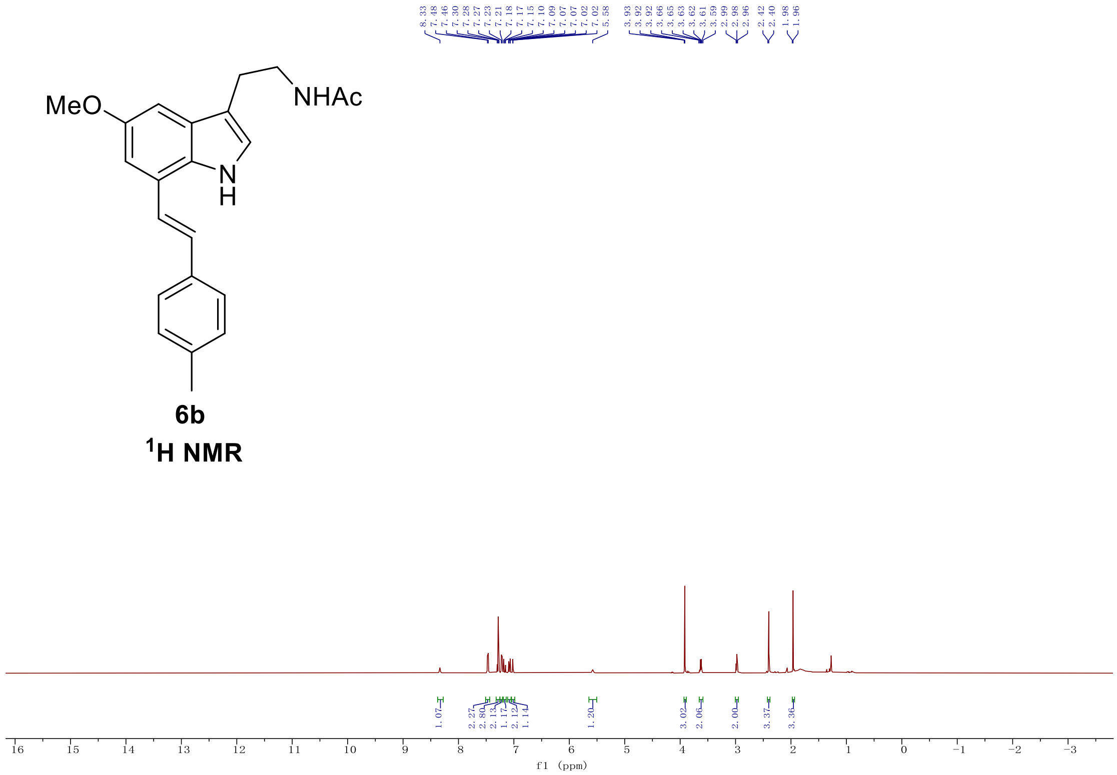
8.33
7.48
7.46
7.30
7.28
7.27
7.23
7.21
7.18
7.17
7.15
7.10
7.09
7.07
7.07
7.02
7.02
5.58
3.93
3.92
3.92
3.66
3.65
3.63
3.62
3.61
3.59
2.99
2.98
2.96
2.42
2.40
1.98
1.96
MeO
NHAc
N
H
6b
1H NMR
1.07
2.27
2.80
2.13
1.17
2.12
1.14
1.20
3.02
2.06
2.00
3.37
3.36
f1 (ppm)

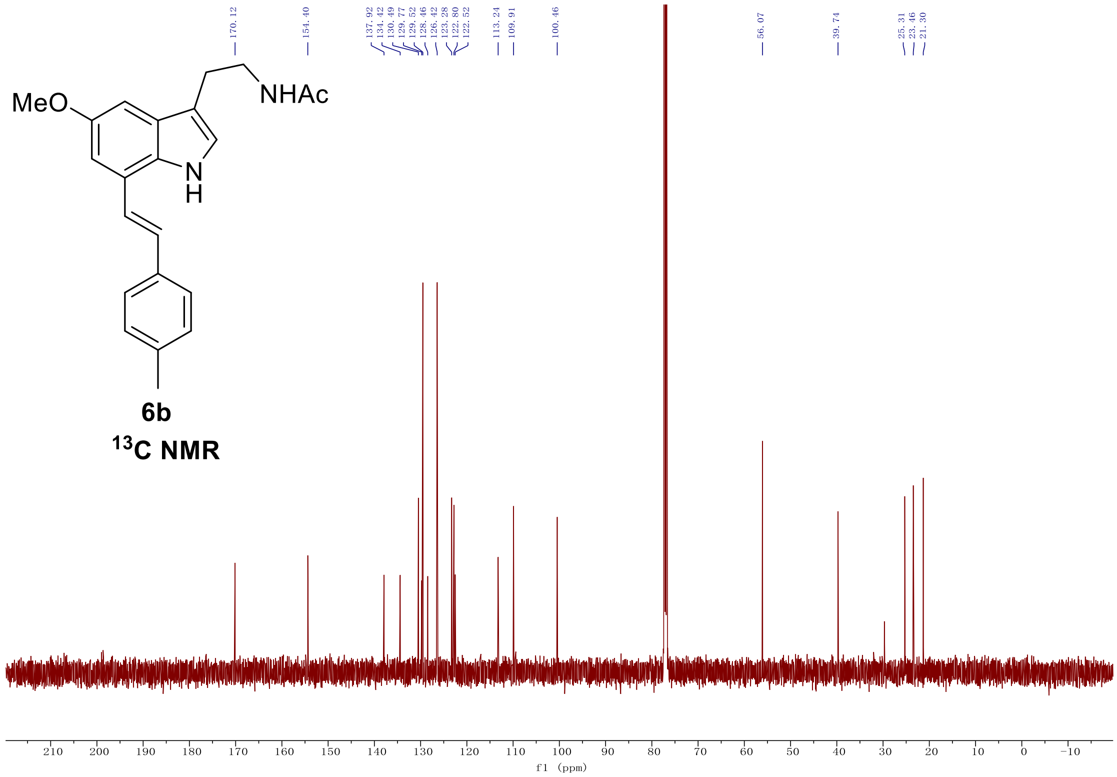
MeO
NHAc
N
H
6b
13C NMR
f1 (ppm)

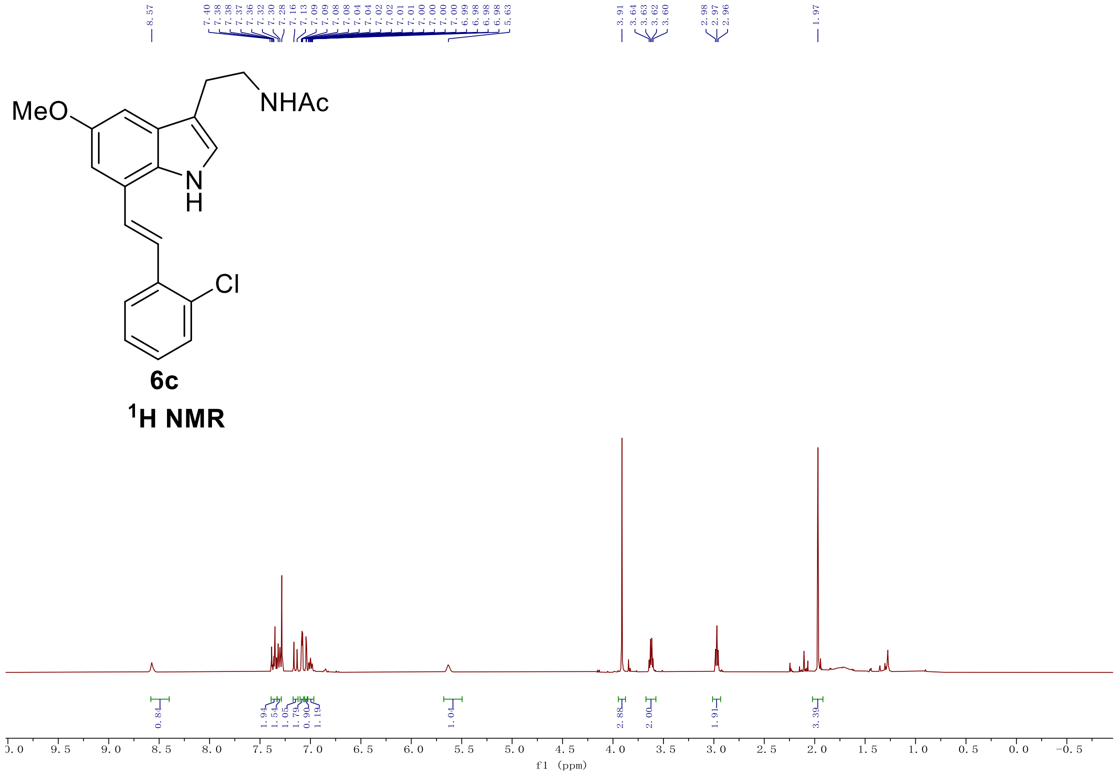
MeO
NHAc
N
H
Cl
6c
1H NMR
f1 (ppm)

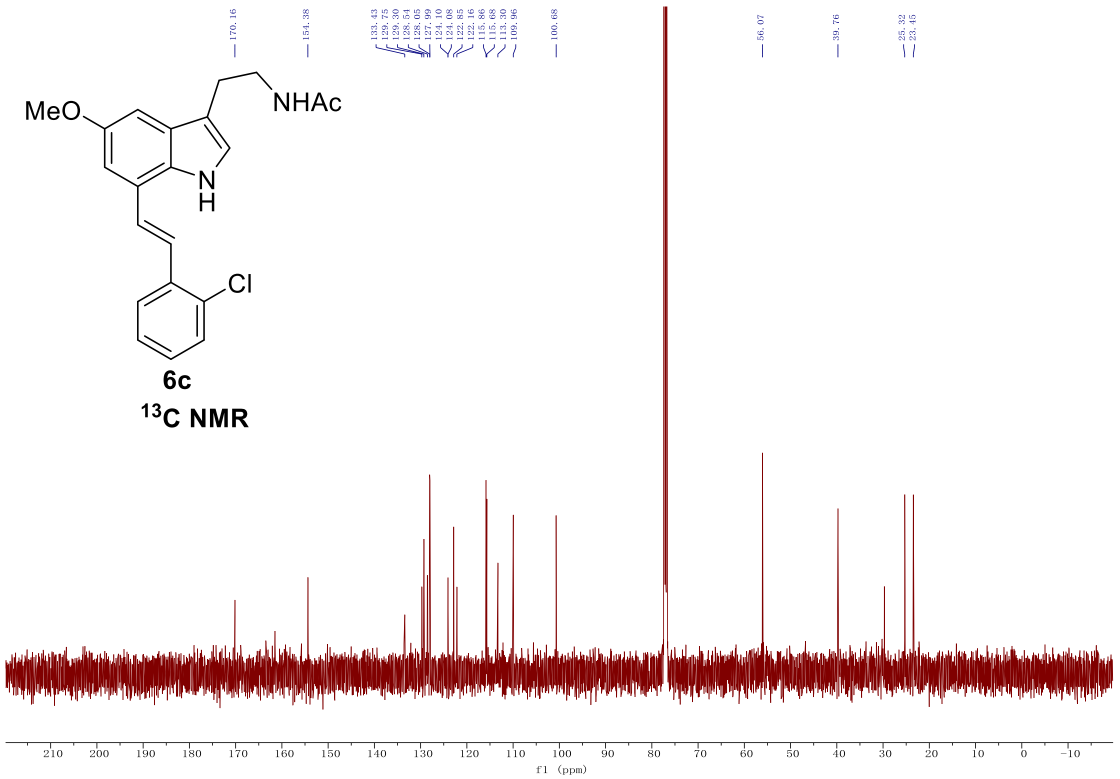
170.16
154.38
133.43
129.75
129.30
128.54
128.05
127.99
124.10
124.08
122.85
122.16
115.86
115.68
113.30
109.96
100.68
56.07
39.76
25.32
23.45
MeO
NHAc
N
H
Cl
6c
13C NMR
f1 (ppm)

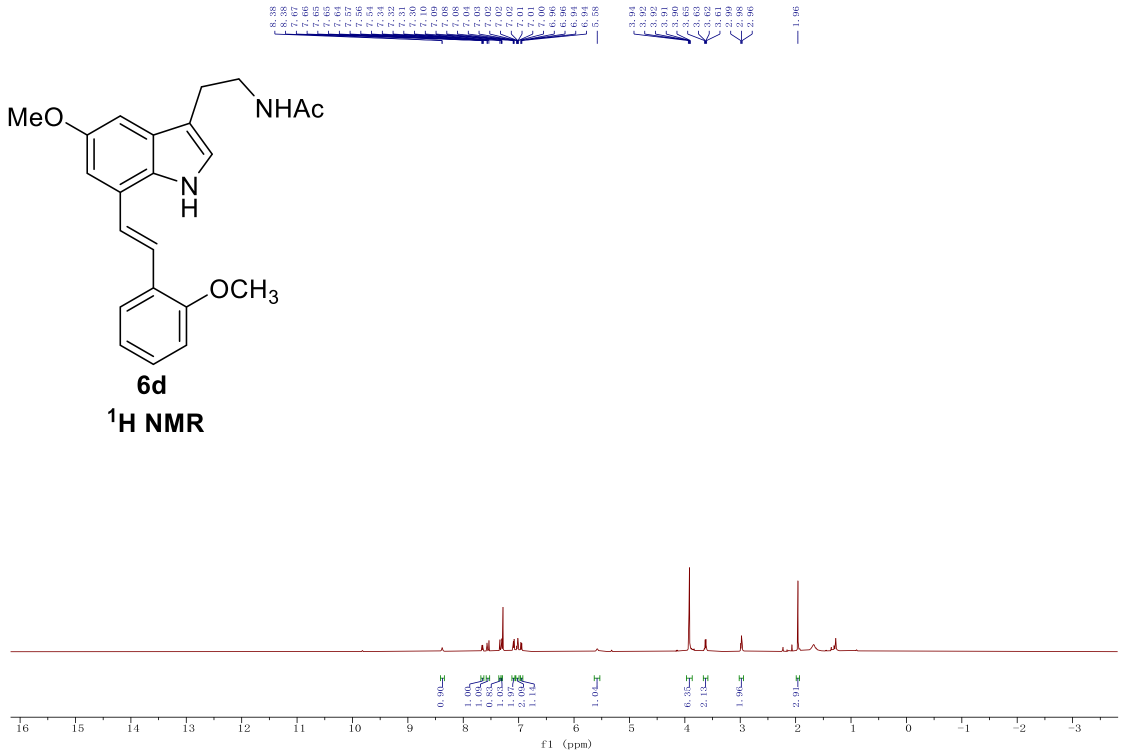
8.38
8.38
7.67
7.66
7.65
7.65
7.64
7.57
7.56
7.54
7.34
7.32
7.31
7.30
7.10
7.09
7.08
7.08
7.04
7.03
7.02
7.02
7.01
7.01
7.00
6.96
6.96
6.94
6.94
5.58
3.94
3.92
3.92
3.91
3.90
3.65
3.63
3.62
3.61
2.99
2.98
2.96
1.96
MeO
NHAc
N
H
OCH3
6d
1H NMR
0.90
1.00
1.09
0.83
1.03
1.97
2.09
1.14
1.04
6.35
2.13
1.96
2.91
f1 (ppm)

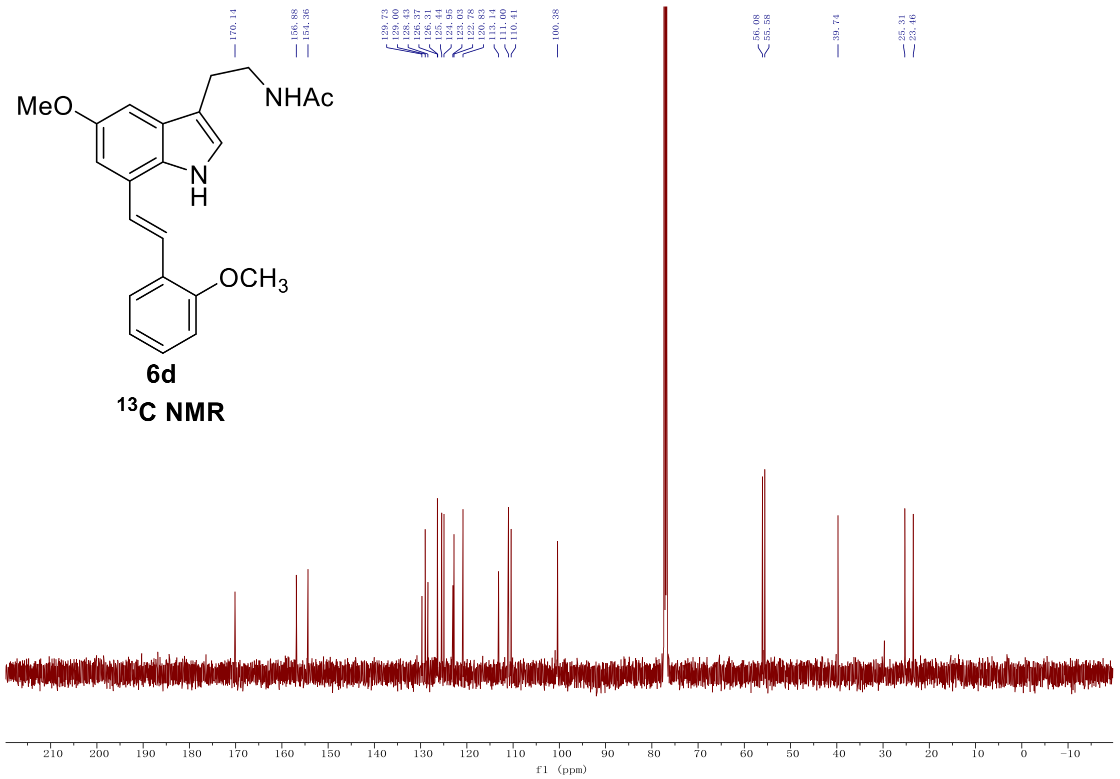

6d

^{13}C NMR

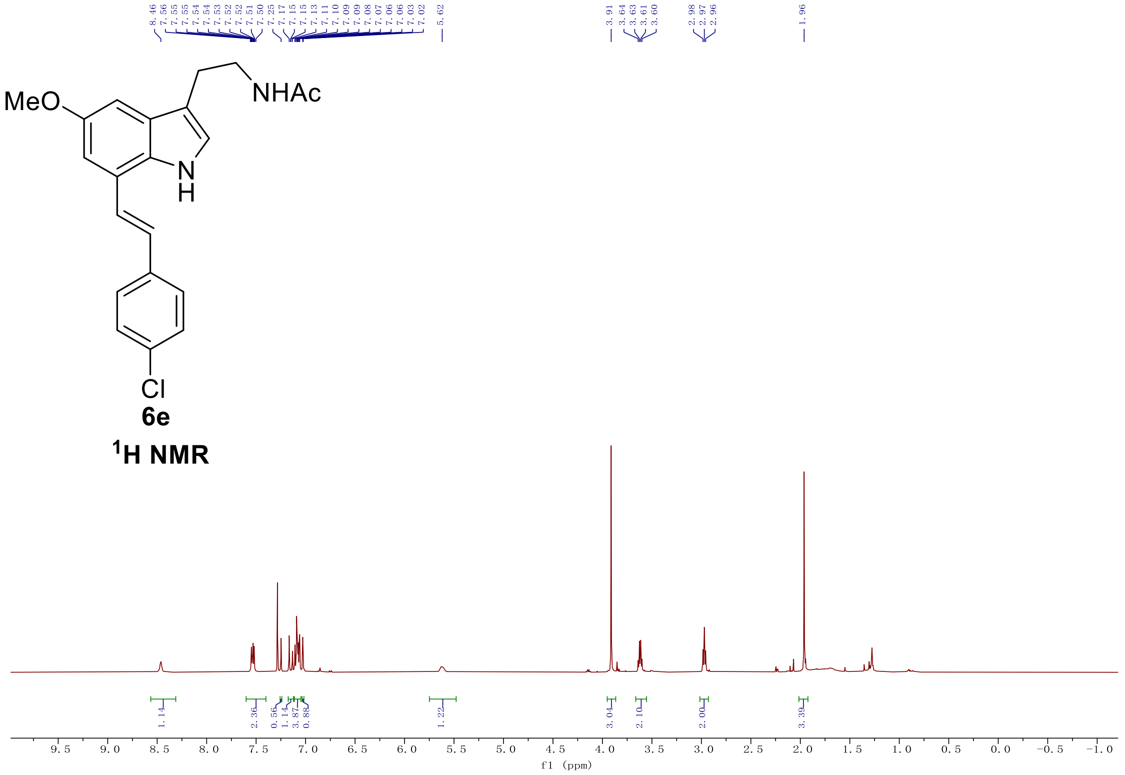

6e

^{1}H NMR

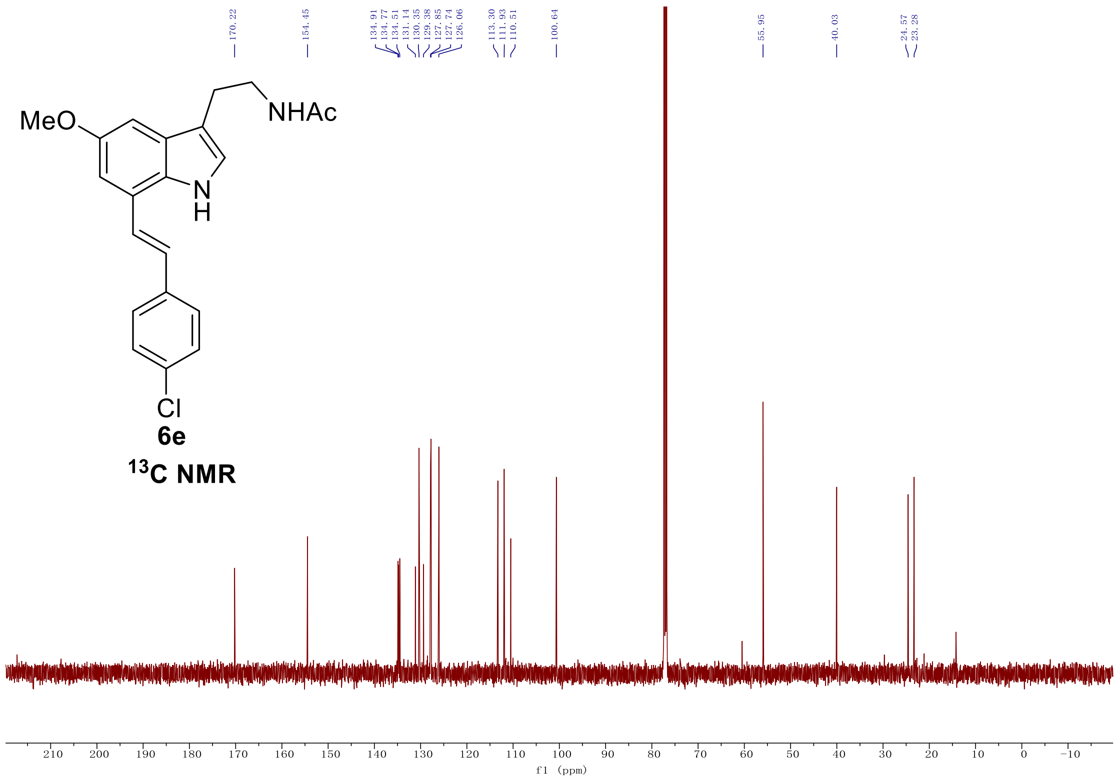
170.22
154.45
134.91
134.77
134.51
131.14
130.35
129.38
127.85
127.74
126.06
113.30
111.93
110.51
100.64
55.95
40.03
24.57
23.28
MeO
NHAc
N
H
Cl
6e
13C NMR
f1 (ppm)

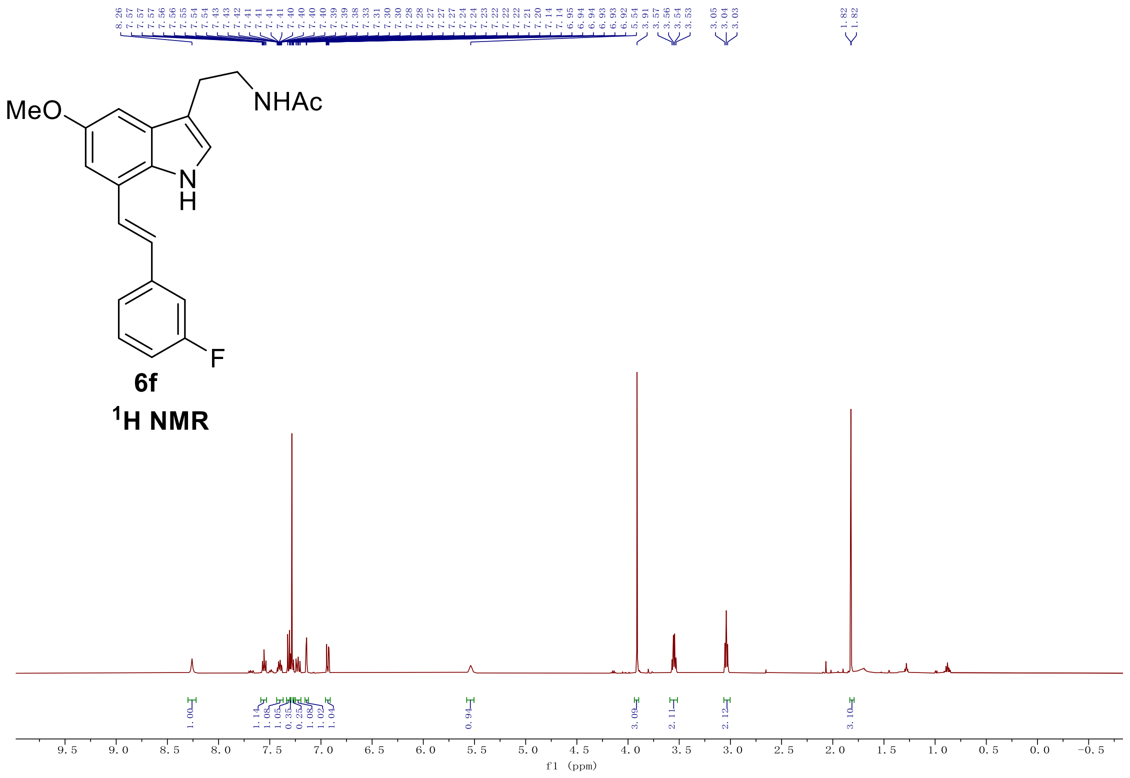
MeO
NHAc
N
H
F
6f
1H NMR
1.00
0.94
3.09
2.11
2.12
3.10
f1 (ppm)

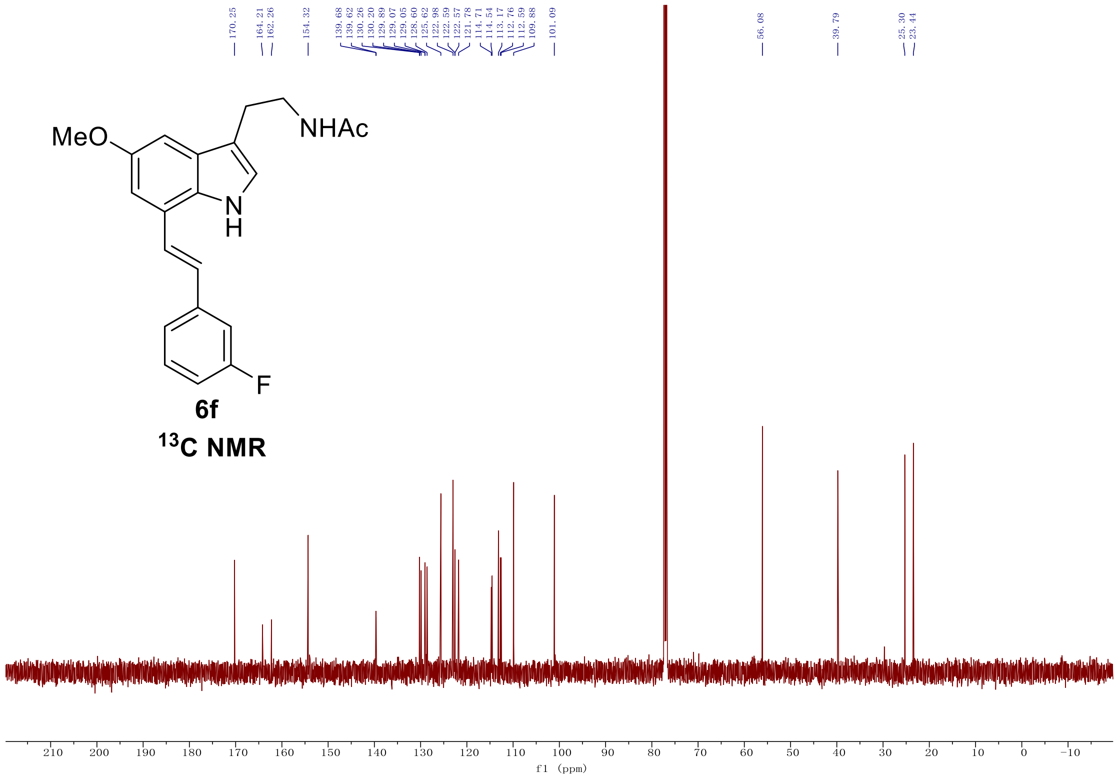

170.25
164.21
162.26
154.32
139.68
139.62
130.26
130.20
129.89
129.07
129.05
128.60
125.62
122.98
122.59
122.57
121.78
114.71
114.54
113.17
112.76
112.59
109.88
101.09
56.08
39.79
25.30
23.44
MeO
NHAc
N
H
F
6f
13C NMR
f1 (ppm)

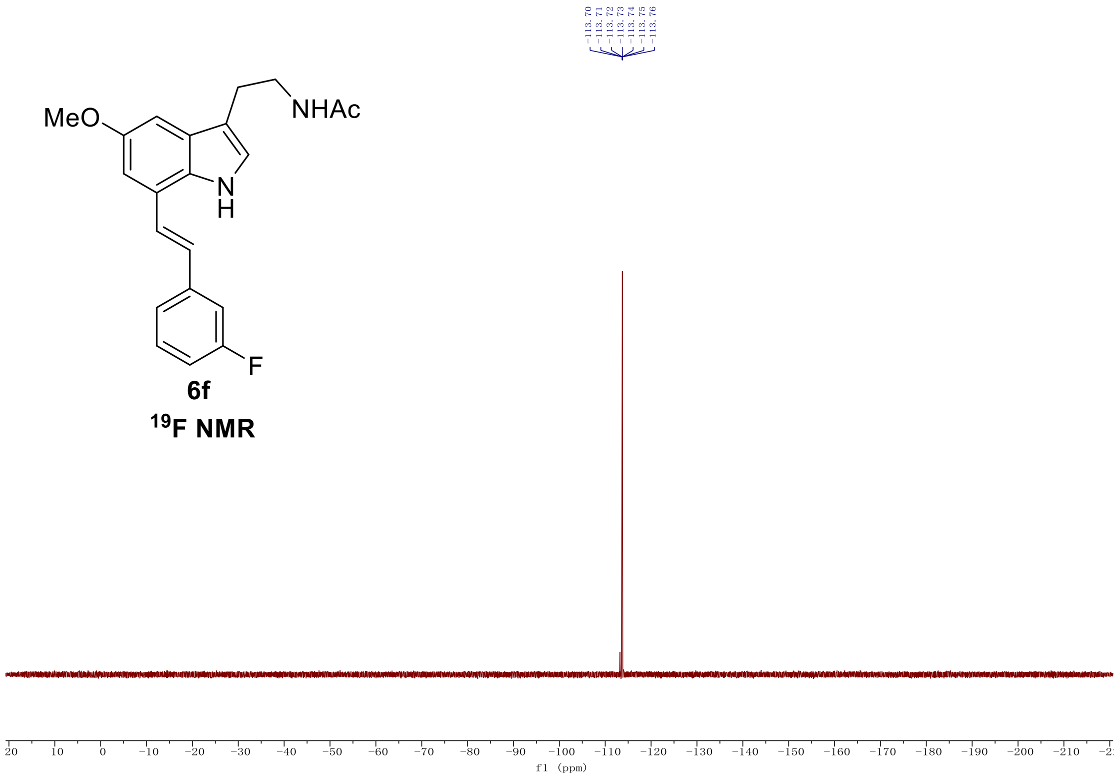

-113.70
-113.71
-113.72
-113.73
-113.74
-113.75
-113.76
MeO
NHAc
N
H
F
6f
19F NMR
f1 (ppm)

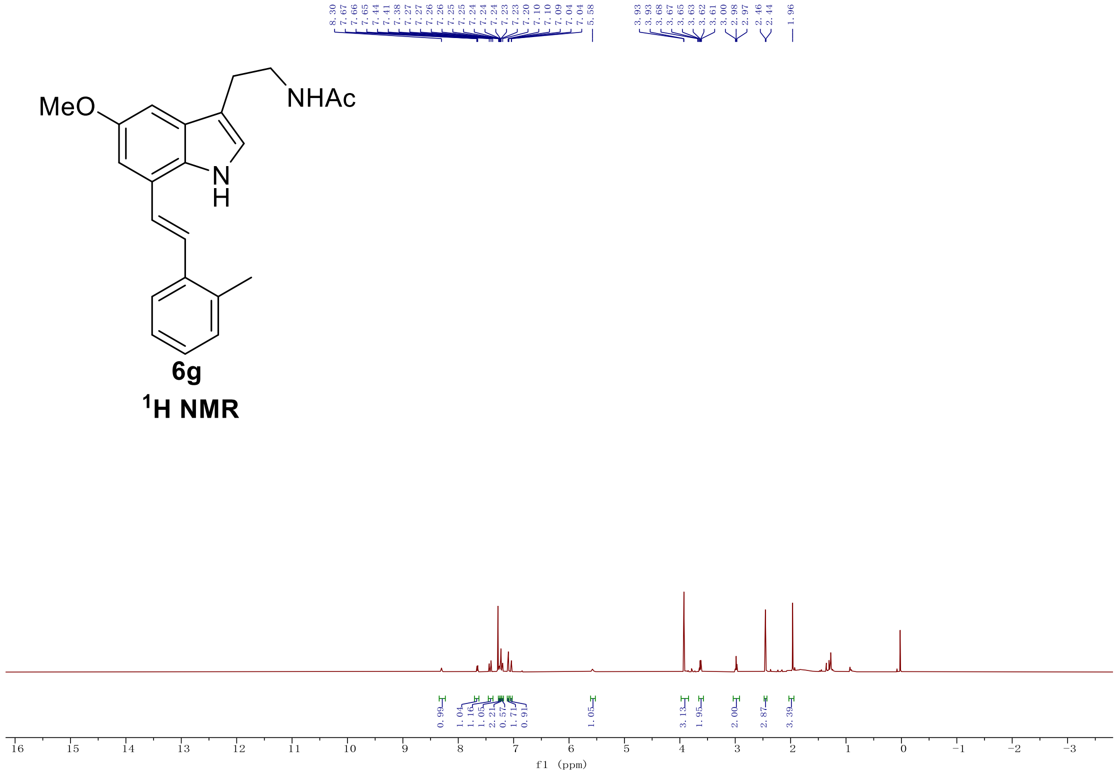

6g

^{1}H NMR

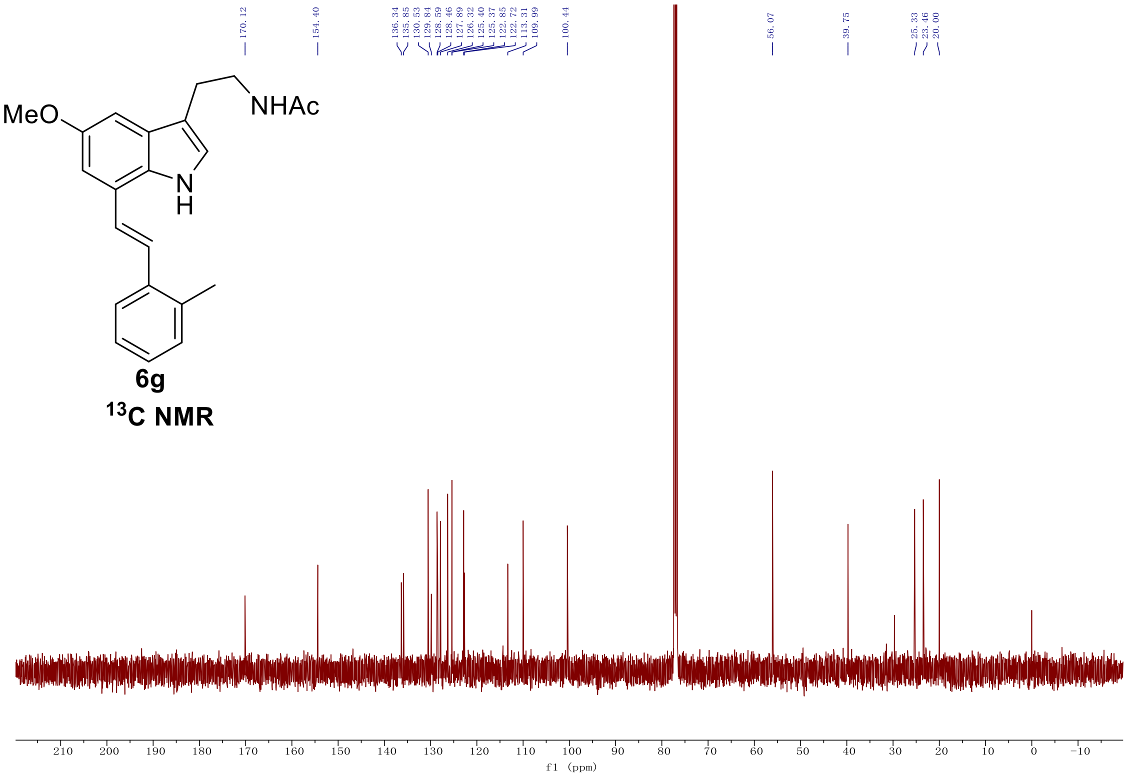

6g

^{13}C NMR

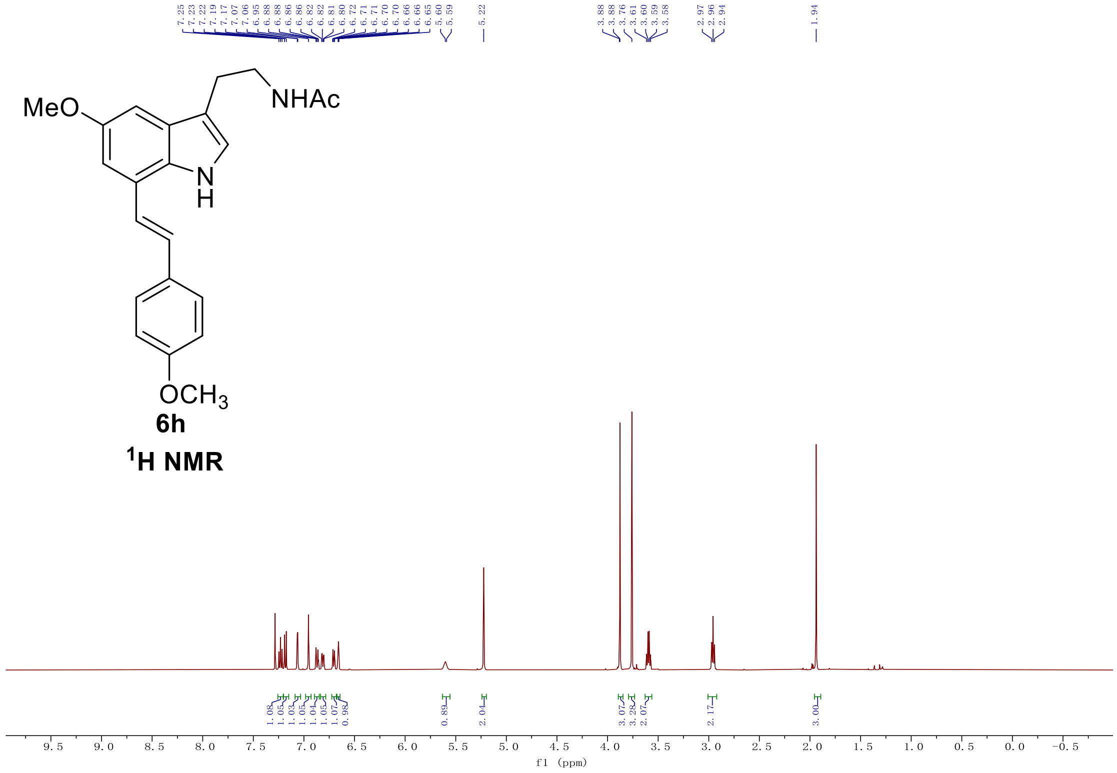
MeO
NHAc
N
H
OCH3
6h
1H NMR
f1 (ppm)

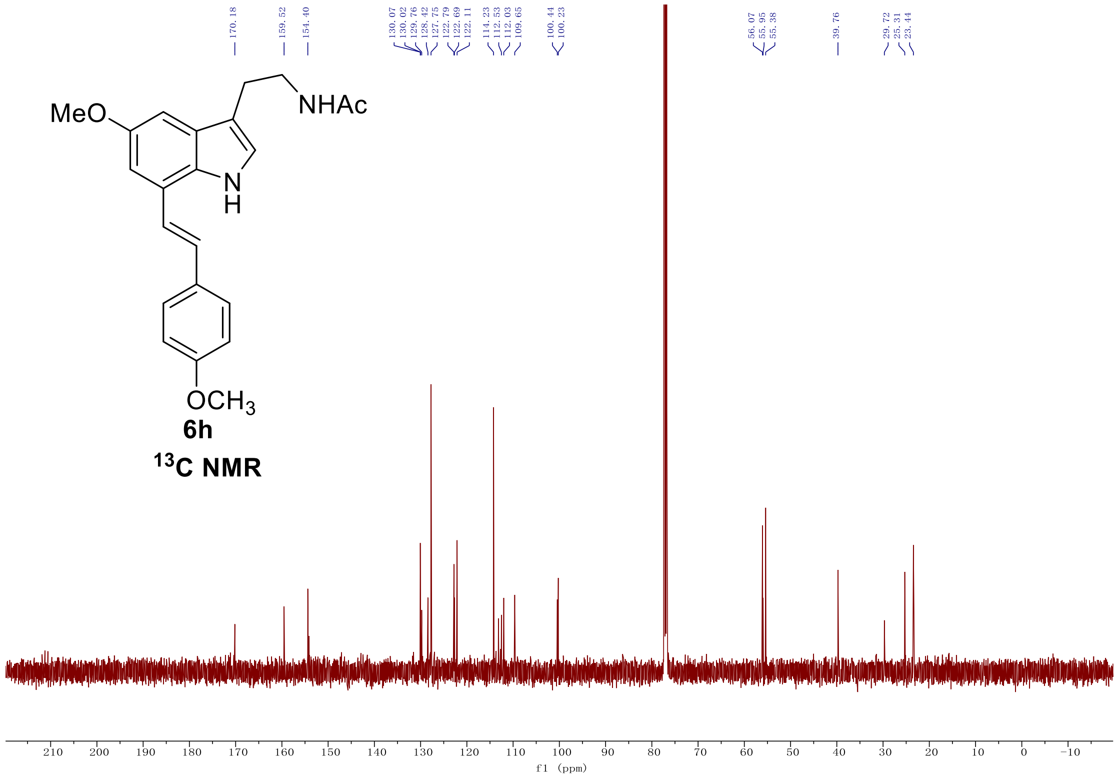
MeO
NHAc
N
H
OCH3
6h
13C NMR
f1 (ppm)

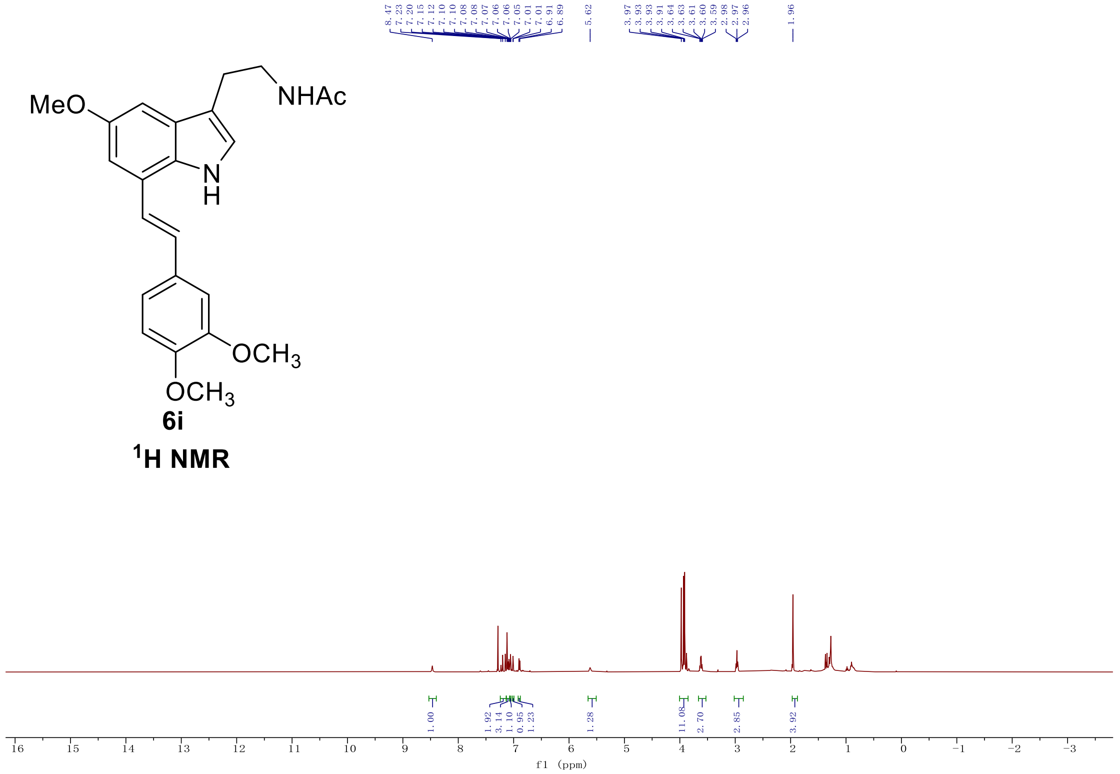
8.47 7.23 7.20 7.15 7.12 7.10 7.10 7.08 7.08 7.07 7.06 7.06 7.05 7.01 7.01 6.91 6.89
5.62
3.97 3.93 3.93 3.91 3.64 3.63 3.61 3.60 3.59 2.98 2.97 2.96
1.96
MeO
NHAc
N
H
OCH3
OCH3
6i
1H NMR
1.00
1.92 3.14 1.10 0.95 1.23
1.28
11.08 2.70 2.85 3.92
f1 (ppm)

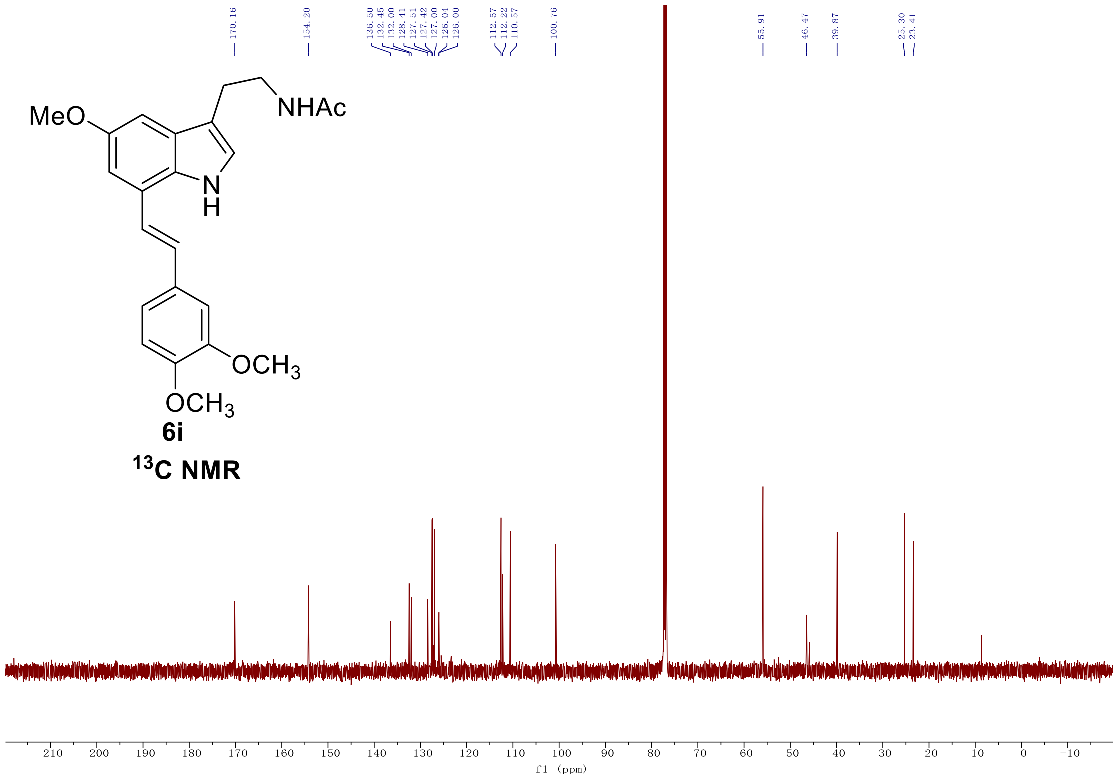
170.16
154.20
136.50 132.45 132.00 128.41 127.51 127.42 127.00 126.04 126.00
112.57 112.22 110.57
100.76
55.91
46.47
39.87
25.30 23.41
MeO
NHAc
N
H
OCH3
OCH3
6i
13C NMR
f1 (ppm)

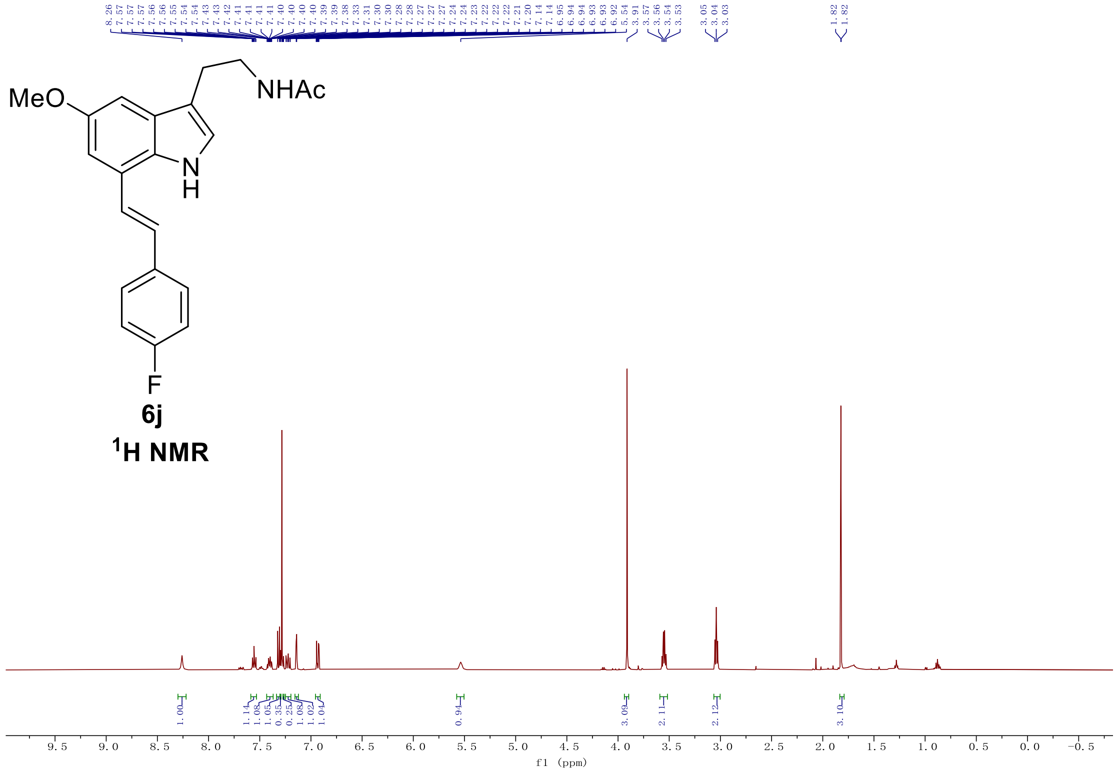
8.26 7.57 7.57 7.56 7.56 7.55 7.54 7.54 7.43 7.43 7.42 7.41 7.41 7.41 7.40 7.40 7.40 7.39 7.39 7.38 7.33 7.31 7.30 7.30 7.28 7.28 7.27 7.27 7.24 7.24 7.23 7.22 7.22 7.21 7.20 7.14 7.14 6.95 6.94 6.94 6.93 6.93 6.92 5.54 5.54 3.91 3.57 3.56 3.54 3.53 3.05 3.04 3.03 1.82 1.82
MeO
NHAc
N
H
F
6j
1H NMR
1.00 1.14 1.08 1.05 0.35 0.25 1.08 1.02 1.04 0.94 3.09 2.11 2.12 3.10
9.5 9.0 8.5 8.0 7.5 7.0 6.5 6.0 5.5 5.0 4.5 4.0 3.5 3.0 2.5 2.0 1.5 1.0 0.5 0.0 -0.5
f1 (ppm)

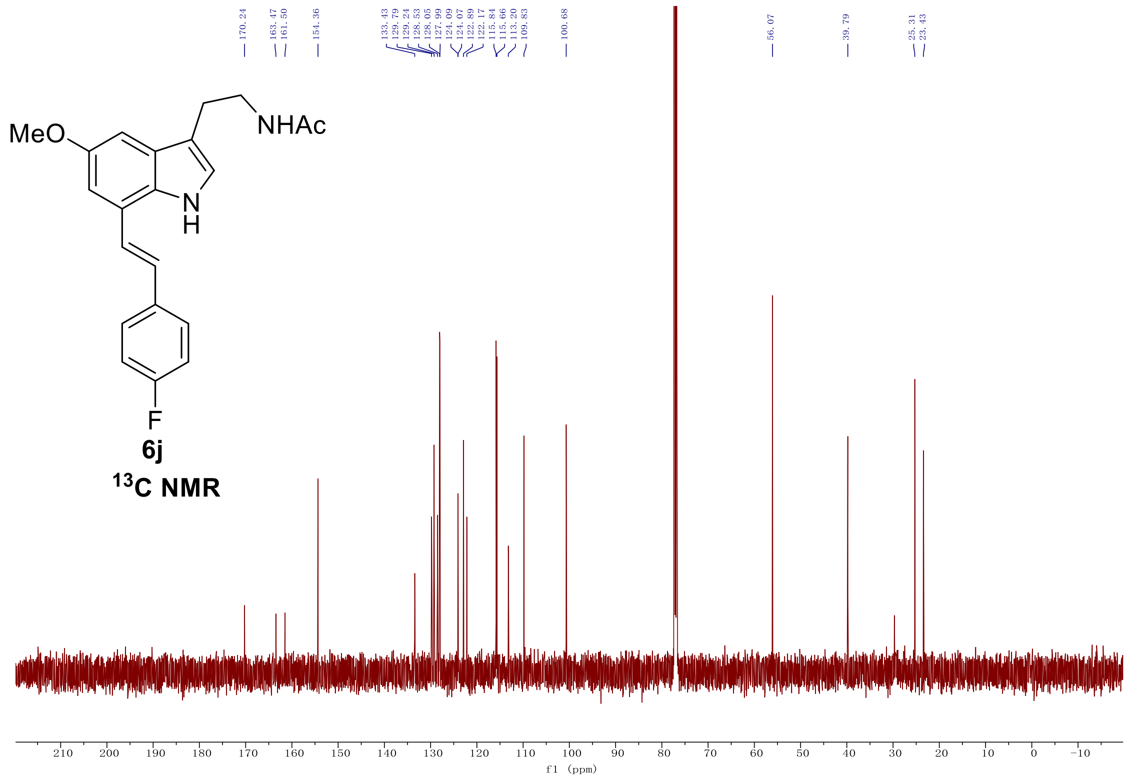
170.24 163.47 161.50 154.36 133.43 129.79 129.24 128.53 128.05 127.99 124.09 124.07 122.89 122.17 115.84 115.66 113.20 109.83 100.68 56.07 39.79 25.31 23.43
MeO
NHAc
N
H
F
6j
13C NMR
210 200 190 180 170 160 150 140 130 120 110 100 90 80 70 60 50 40 30 20 10 0 -10
f1 (ppm)

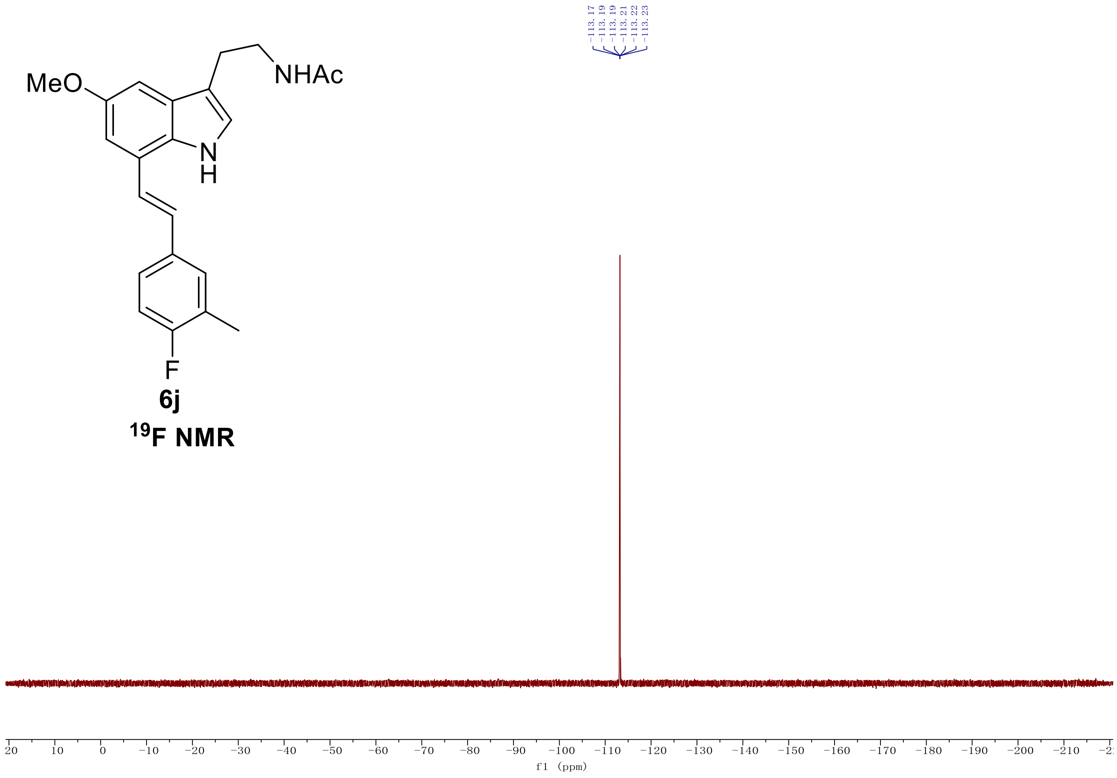

5. Mass Spectrometry Data

3

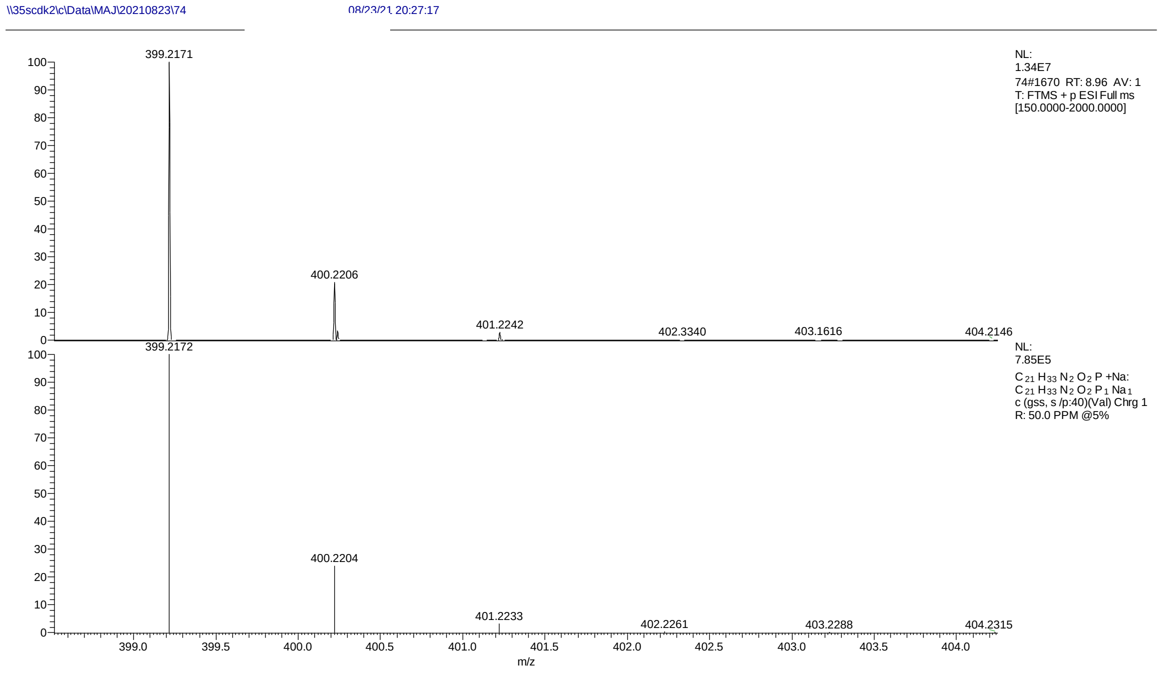

6a

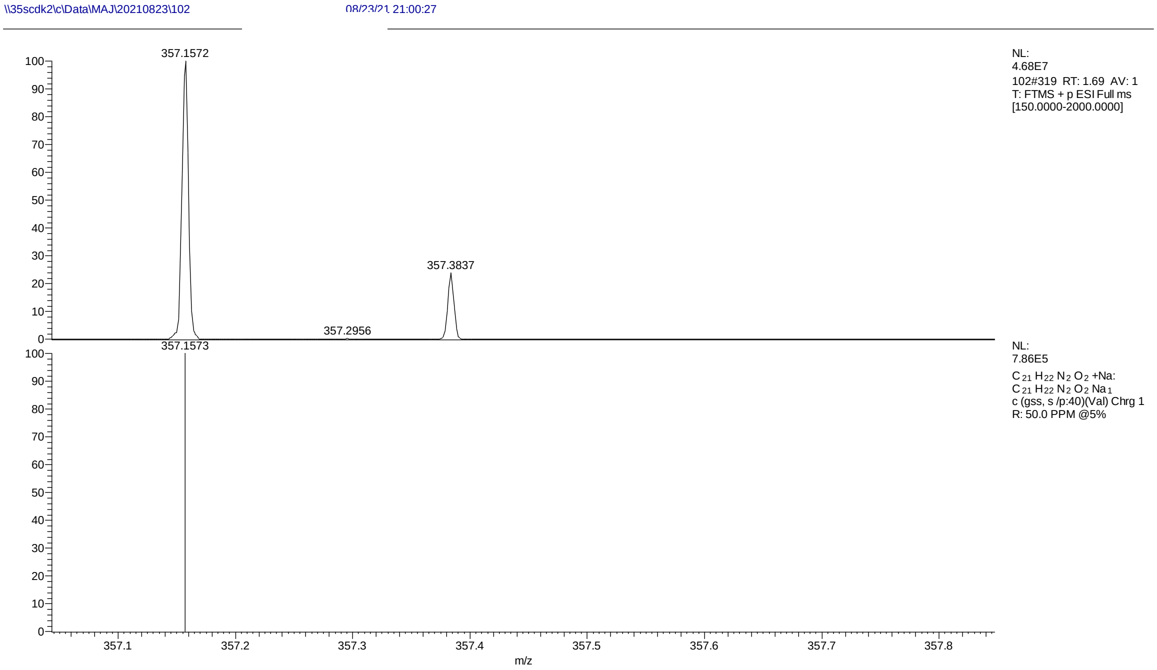
\\35scdk2\c\Data\MAJ\20210823\102
08/23/21 21:00:27
357.1572
357.3837
357.2956
357.1573
NL:
4.68E7
102#319 RT: 1.69 AV: 1
T: FTMS + p ESI Full ms
[150.0000-2000.0000]
NL:
7.86E5
C 21 H22 N2 O2 +Na:
C 21 H22 N2 O2 Na1
c (gss, s /p:40)(Val) Chrg 1
R: 50.0 PPM @5%
m/z

6b

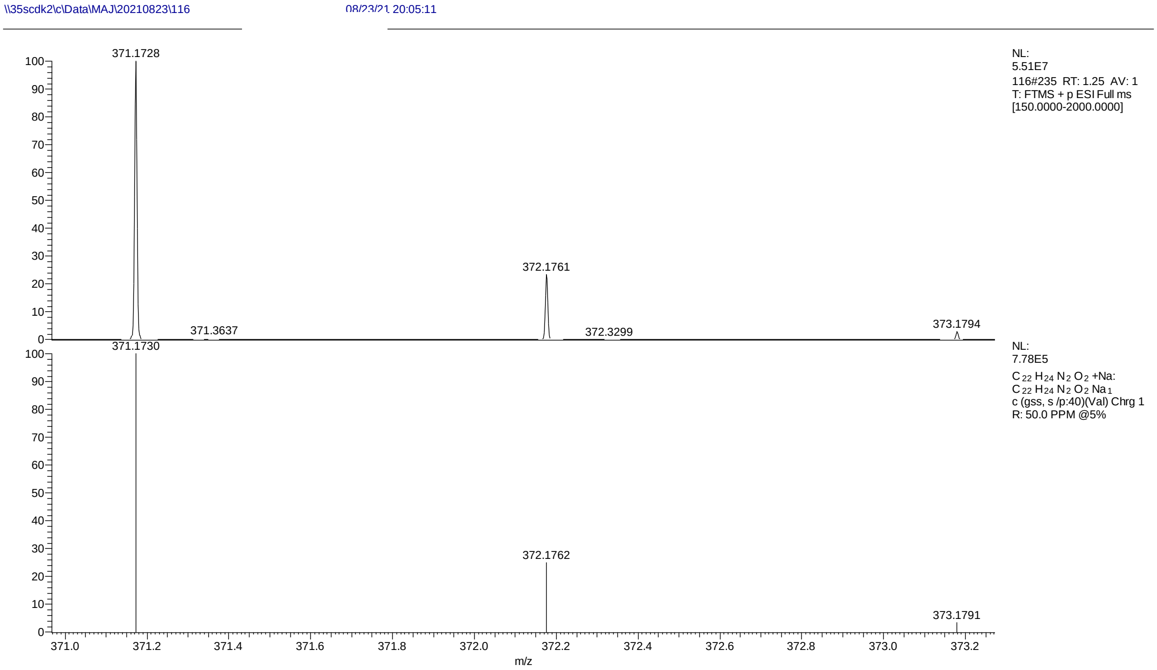
\\35scdk2\c\Data\MAJ\20210823\116
08/23/21 20:05:11
371.1728
371.3637
372.1761
372.3299
373.1794
371.1730
372.1762
373.1791
NL:
5.51E7
116#235 RT: 1.25 AV: 1
T: FTMS + p ESI Full ms
[150.0000-2000.0000]
NL:
7.78E5
C 22 H24 N2 O2 +Na:
C 22 H24 N2 O2 Na1
c (gss, s /p:40)(Val) Chrg 1
R: 50.0 PPM @5%
m/z

6c

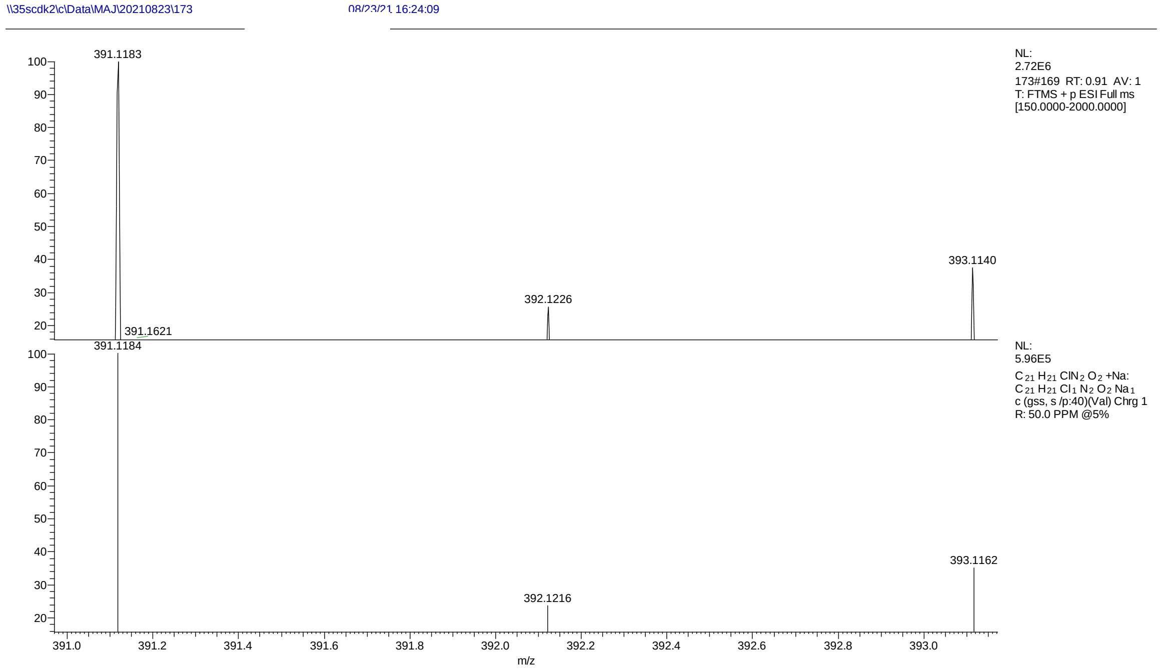

\\35scdk2\c\Data\MAJ\20210823\173
08/23/21 16:24:09
391.1183
391.1621
392.1226
393.1140
NL:
2.72E6
173#169 RT: 0.91 AV: 1
T: FTMS + p ESI Full ms
[150.0000-2000.0000]
391.1184
392.1216
393.1162
NL:
5.96E5
C 21 H21 ClN 2 O2 +Na:
C 21 H21 Cl1 N2 O2 Na 1
c (gss, s /p:40)(Val) Chrg 1
R: 50.0 PPM @5%
m/z

6d

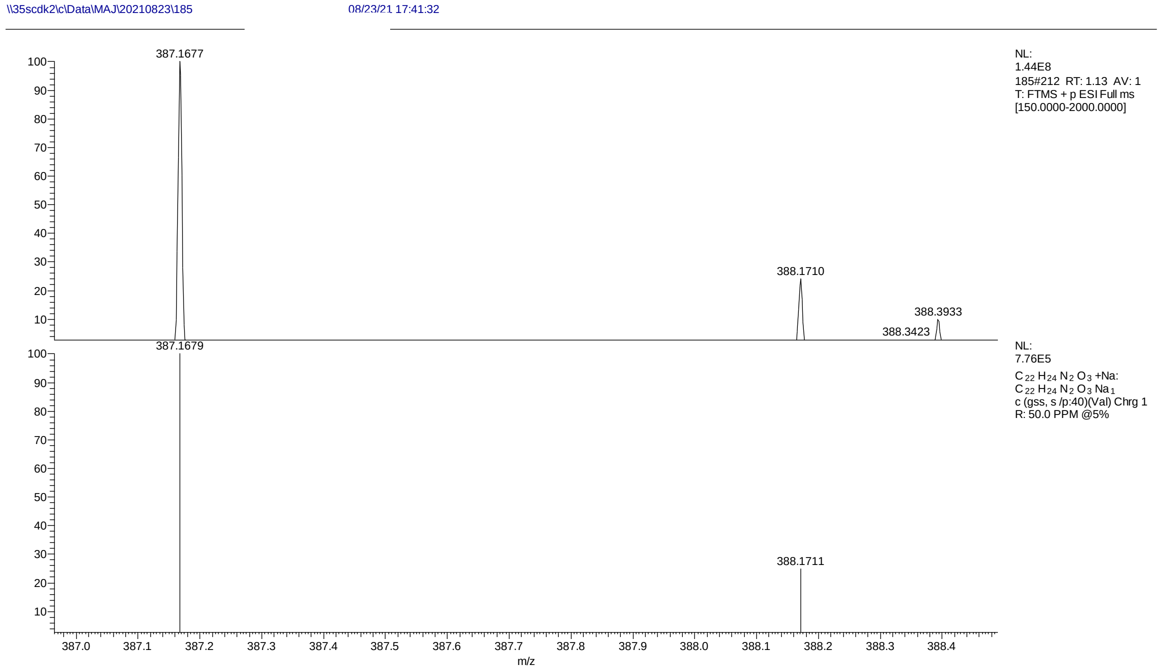

\\35scdk2\c\Data\MAJ\20210823\185
08/23/21 17:41:32
387.1677
388.1710
388.3423
388.3933
NL:
1.44E8
185#212 RT: 1.13 AV: 1
T: FTMS + p ESI Full ms
[150.0000-2000.0000]
387.1679
388.1711
NL:
7.76E5
C 22 H24 N2 O3 +Na:
C 22 H24 N2 O3 Na 1
c (gss, s /p:40)(Val) Chrg 1
R: 50.0 PPM @5%
m/z

6e

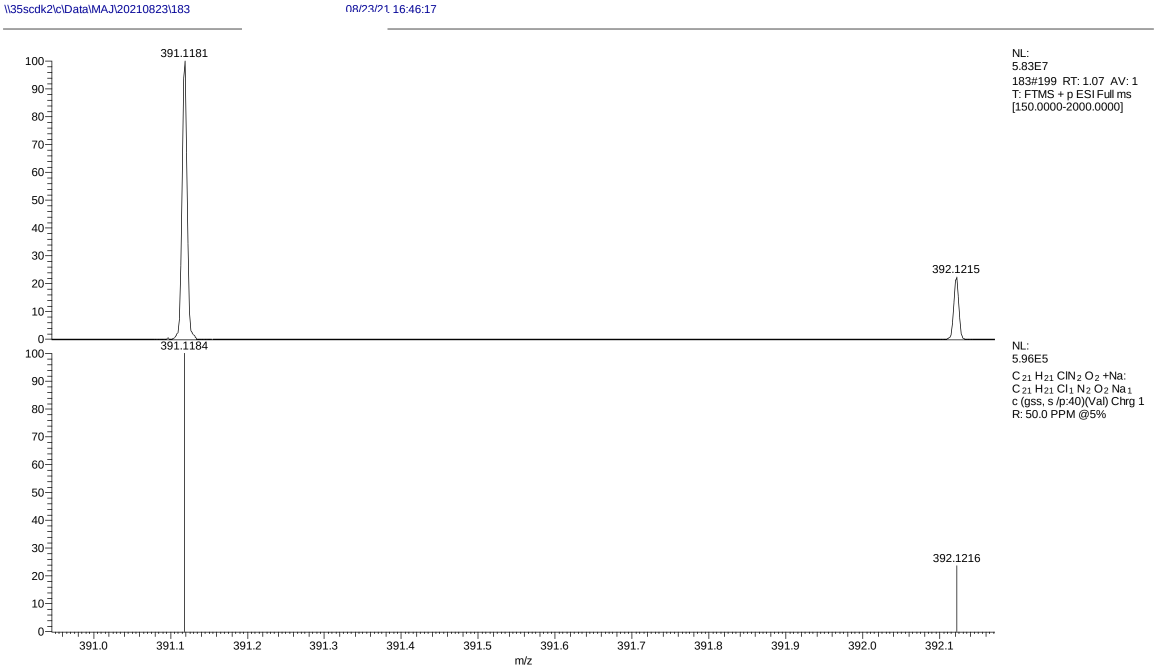
\\35scdk2\c\Data\MAJ\20210823\183
08/23/21 16:46:17
391.1181
392.1215
NL:
5.83E7
183#199 RT: 1.07 AV: 1
T: FTMS + p ESI Full ms
[150.0000-2000.0000]
391.1184
392.1216
NL:
5.96E5
C 21 H21 ClN 2 O2 +Na:
C 21 H21 Cl1 N2 O2 Na 1
c (gss, s /p:40)(Val) Chrg 1
R: 50.0 PPM @5%
m/z

6f

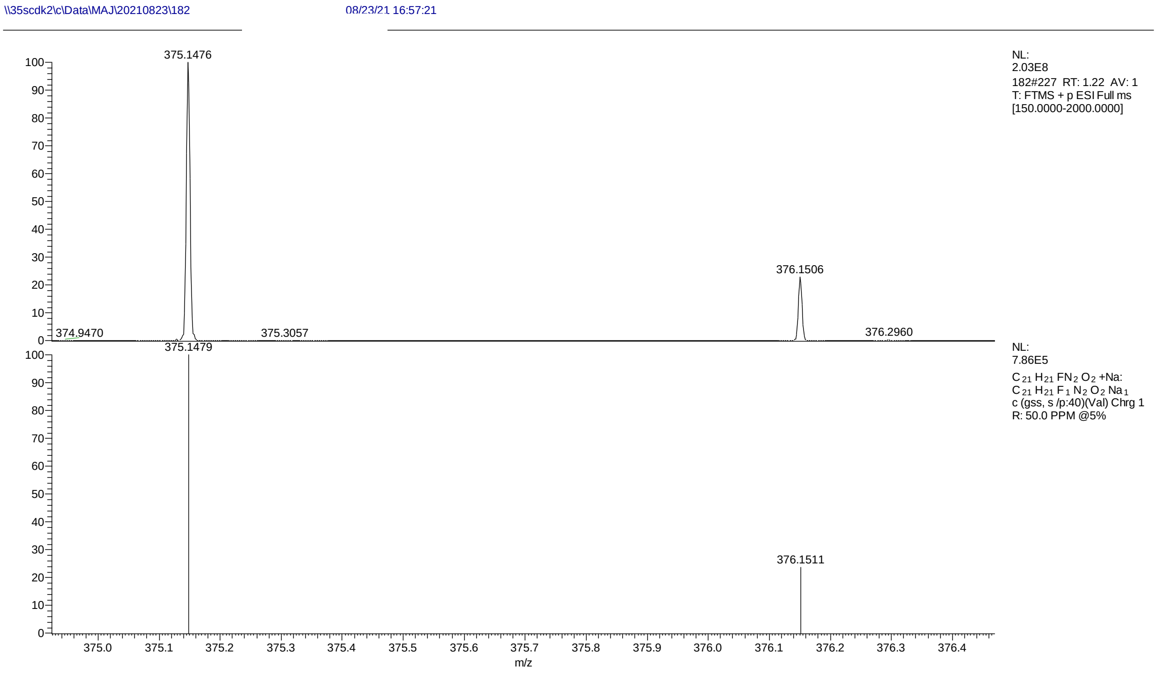
\\35scdk2\c\Data\MAJ\20210823\182
08/23/21 16:57:21
375.1476
376.1506
374.9470
375.3057
376.2960
NL:
2.03E8
182#227 RT: 1.22 AV: 1
T: FTMS + p ESI Full ms
[150.0000-2000.0000]
375.1479
376.1511
NL:
7.86E5
C 21 H21 FN 2 O2 +Na:
C 21 H21 F1 N2 O2 Na 1
c (gss, s /p:40)(Val) Chrg 1
R: 50.0 PPM @5%
m/z

6g

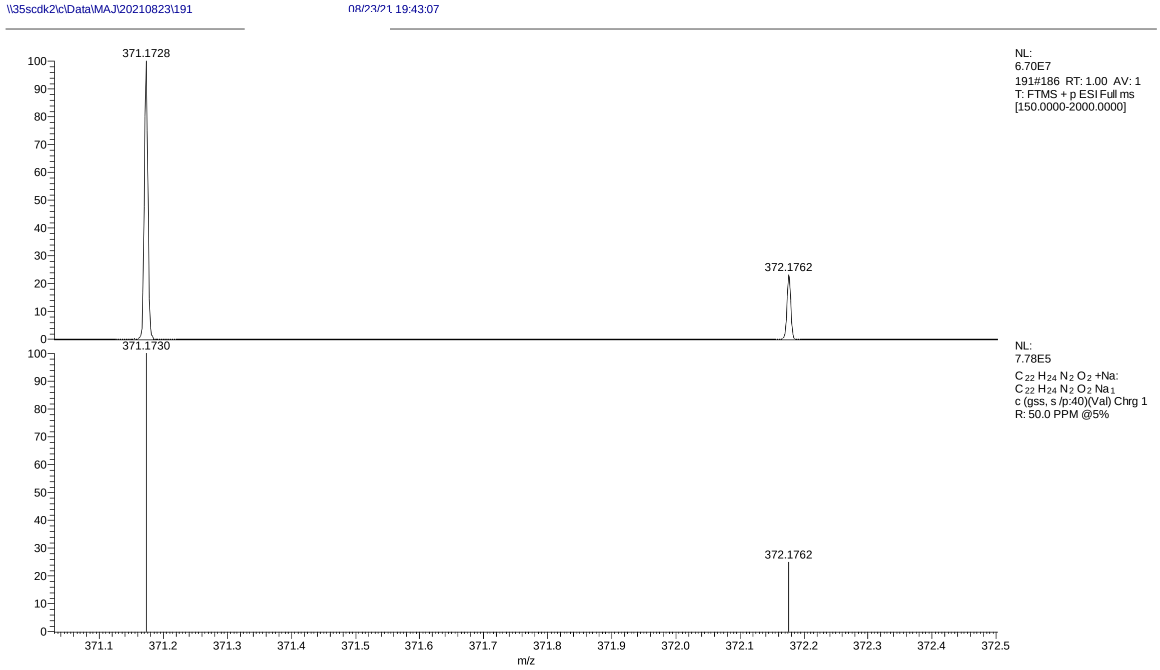
\\35scdk2\c\Data\MAJ\20210823\191
08/23/21 19:43:07
371.1728
372.1762
NL:
6.70E7
191#186 RT: 1.00 AV: 1
T: FTMS + p ESI Full ms
[150.0000-2000.0000]
371.1730
372.1762
NL:
7.78E5
C 22 H24 N2 O2 +Na:
C 22 H24 N2 O2 Na1
c (gss, s /p:40)(Val) Chrg 1
R: 50.0 PPM @5%
m/z

6h

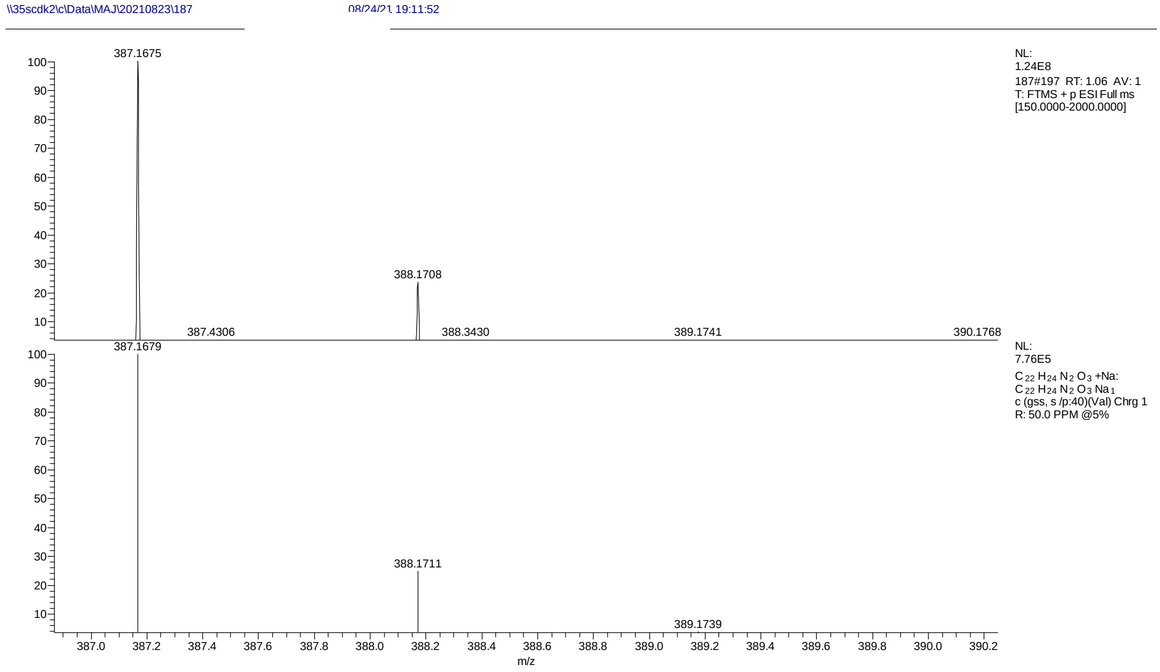
\\35scdk2\c\Data\MAJ\20210823\187
08/24/21 19:11:52
387.1675
388.1708
387.4306
388.3430
389.1741
390.1768
NL:
1.24E8
187#197 RT: 1.06 AV: 1
T: FTMS + p ESI Full ms
[150.0000-2000.0000]
387.1679
388.1711
389.1739
NL:
7.76E5
C 22 H24 N2 O3 +Na:
C 22 H24 N2 O3 Na1
c (gss, s /p:40)(Val) Chrg 1
R: 50.0 PPM @5%
m/z

6i

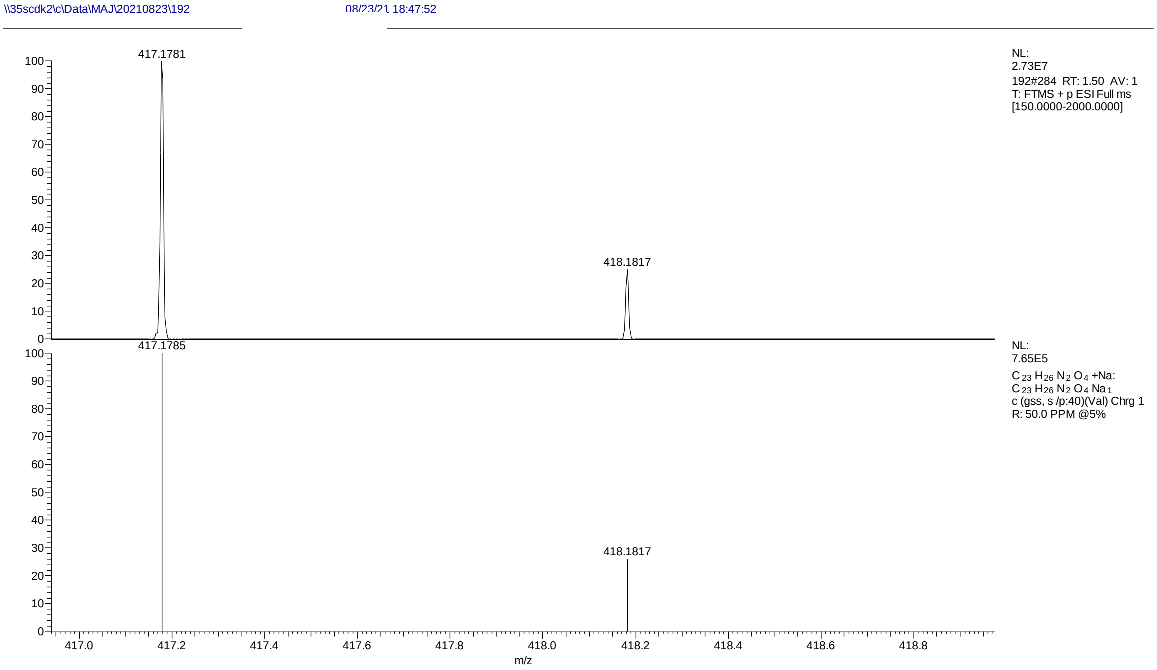
\\35scdk2\c\Data\MAJ\20210823\192
08/23/21 18:47:52
417.1781
418.1817
NL:
2.73E7
192#284 RT: 1.50 AV: 1
T: FTMS + p ESI Full ms
[150.0000-2000.0000]
417.1785
418.1817
NL:
7.65E5
C 23 H 26 N 2 O 4 +Na:
C 23 H 26 N 2 O 4 Na 1
c (gss, s /p:40)(Val) Chrg 1
R: 50.0 PPM @5%
m/z

6j

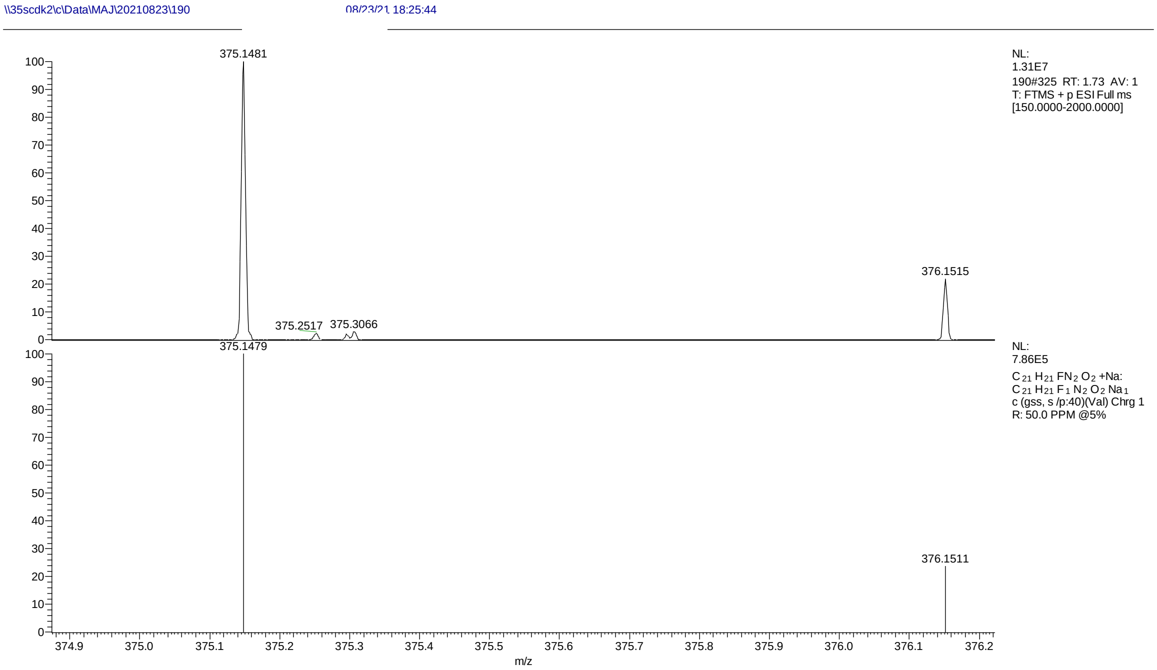
\\35scdk2\c\Data\MAJ\20210823\190
08/23/21 18:25:44
375.1481
375.2517
375.3066
376.1515
NL:
1.31E7
190#325 RT: 1.73 AV: 1
T: FTMS + p ESI Full ms
[150.0000-2000.0000]
375.1479
376.1511
NL:
7.86E5
C 21 H 21 FN 2 O 2 +Na:
C 21 H 21 F 1 N 2 O 2 Na 1
c (gss, s /p:40)(Val) Chrg 1
R: 50.0 PPM @5%
m/z
